# Cultivation and sequencing of microbiota members unveil the functional potential of yak gut microbiota

**DOI:** 10.1128/msystems.00367-25

**Published:** 2025-08-15

**Authors:** Min Dai, Fangfang Zhao, Xingwei Shi, Chen Tian, Yuheng Lin, Liyan Bai, Teng Li, Xin Jin, Liang Xiao, Karsten Kristiansen, Xiaoping Li, Zhigang Zhang

**Affiliations:** 1 College of Life Sciences, University of Chinese Academy of Sciences 617066 , Beijing, China; 2 BGI Research 734174, Wuhan, Hubei, China; 3 State Key Laboratory for Conservation and Utilization of Bio-Resources in Yunnan, School of Life Sciences, Yunnan University 674423 , Kunming, Yunnan, China; 4 Gansu Key Laboratory of Herbivorous Animal Biotechnology, Faculty of Animal Science and Technology, Gansu Agricultural University 454423, Lanzhou, Gansu, China; 5 BGI Research, Shenzhen, China; 6 Shenzhen Engineering Laboratory of Detection and Intervention of Human Intestinal Microbiome, BGI Research, Shenzhen, China; 7 Laboratory of Integrative Biomedicine, Department of Biology, University of Copenhagen 56603, Copenhagen, Denmark; 8 Shenzhen Key Laboratory of Environmental Microbial Genomics and Application, BGI Research, Shenzhen, China; Chinese Academy of Sciences, Beijing, China

**Keywords:** ruminants, gut microbiota, potential resource

## Abstract

The animal gut microbiota exhibits extensive taxonomic diversity, yet cultivated isolates and complete genomes from animal hosts remain scarce, hindering functional and ecological insights. We present a cultivated Yak (*Bos grunniens*) Fecal bacteria genome Reference (YFR), comprising 548 high-quality genomes based on aerobic and anaerobic cultivation. Notably, 216 strains represented novel taxa, classified into 29 species-level clusters spanning 4 phyla and 14 genera. The YFR increased the proportion of cultured ruminant gut bacterial species by 19.39%, significantly expanding the reference database for this ecosystem. Among these, 11 species harbor abundant CAZymes Gene Clusters (CGCs), indicating a high capacity for digesting complex polysaccharides. Biosynthetic Gene Clusters (BGCs) are predicted and demonstrated to possess distinct novelty in YFR genomes, demonstrating a potential for future applications. We demonstrated that the symbiotic relationship between host bacterial strains and bacteriophages can be effectively studied using cultured strains by enabling precise mapping of viral genes to host metabolic adaptations. Culturing animal gut bacterial species not only expands the resources of culturable strains but also provides a basis for subsequent functional mining.

**IMPORTANCE:**

As a representative species in high-altitude extreme environments, yaks rely on their gut microbiota to support critical physiological functions and adapt to harsh conditions. This study established a comprehensive pipeline by integrating innovative single-bacterium culture conditions with optimized strategies for the yak gut microbiota. The resulting genomic repository not only expands the culturable microbial resources for extremophile mammals but also reveals unique metabolic traits, including polysaccharide-digesting CAZyme clusters, novel BGCs, and phage-host interactions. This approach provides essential microbial resources for advancing our understanding of host-microbial adaptations to extreme environments and offers tangible tools for industrial enzyme discovery and synthetic biology applications.

## INTRODUCTION

Microbial communities play a pivotal role in the growth of host animals and may assist them in adapting to the external environment. As research on animal gut microbiota advances, a substantial number of potentially novel microbial species with huge untapped functions have been identified (1–3). Metagenomic approaches provide a blueprint of microbial communities in the gut, enhancing our understanding of animal gut microbiota. However, cultivation-dependent studies offer opportunities for better exploration of the diverse functions of microbiota based on information on single strains and complete genome sequences (4). Extensive work on the cultivation and sequencing of human gut bacteria has resulted in the construction of large catalogs of individual bacterial genomes, and to date, over 10,000 microbial genomes of the human microbiome have been characterized (4–10). Isolation and cultivation efforts in mice (11), pigs (12), and cattle (13) have also provided a rich source of bacterial isolates and high-quality genomes.

We have conducted metagenomic investigations of the gut microbiota of animals inhabiting the Qinghai-Tibet Plateau, unveiling a plethora of potentially novel and functionally diverse microorganisms (14). In this study, we focused on yak fecal samples and cultured bacterial strains using four different media in aerobic and anaerobic environments. As a result, we successfully isolated 1,192 bacterial strains, adding 267 new reference genomes to the list of ruminant gut microbes, including a collection of 548 reference genomes of ruminant gut microbes, which were classified into 77 species (15). Our investigation further revealed that these microorganisms exhibit a wide range of functional diversity, which holds interesting potential for future research.

## MATERIALS AND METHODS

### Bacterial culture

Microbial culture samples were derived from fresh feces of 16 grazing yaks in the Nam Co Lake area on the Qinghai-Tibet Plateau. Detailed information is provided in Table S1. Samples were immediately preserved in a proprietary in-house microbial preservation solution (developed to maintain bacterial viability and genomic integrity), transported under controlled conditions (4°C) within 24 h, and stored at −80°C upon arrival at the BGI-Shenzhen laboratory. The samples were diluted to seven concentrations (10^−1^ to 10^−7^) and spread on agar plates for cultivation under aerobic (clean bench, incubator) and anaerobic (Bactron 600-2 anaerobic chamber, gas mixture ratio for cultivation: 90% N_2_, 5% CO_2_, and 5% H_2_) conditions. Four different agar media were used, with specific formulations detailed in Supplementary Material. Incubation was conducted at 37°C for 3–4 days. Selected individual colonies were re-streaked to obtain single-clonal strains and then transferred to the corresponding liquid media for 1–2 days of cultivation, as illustrated in Fig. S1. Strains isolated in aerobic environments were stored in a glycerol suspension (20%, vol/vol) at −80°C, whereas strains isolated in anaerobic environments were stored in a glycerol suspension (20%, vol/vol) containing 0.1% cysteine at −80°C. All bacterial strains were deposited in the China National GeneBank, and the sequencing data are accessible through CNSA CNP0004162.

### 16S rRNA gene amplification and identification

Isolates were grown to the stationary phase and then centrifuged at 9,570 × *g* for 5 min at 4°C. The bacterial pellets were resuspended in phosphate-buffered saline (PBS) at the same volume as the original culture. The V1-V5 region of the 16S rRNA gene was amplified using universal primers (27F: AGAGTTTGATCATGGCTCAG, 806R: GGACTACNVGGGTWTCTAAT) (16, 17). Sanger sequencing was performed by BGI Write (Guangdong, China), as described previously (4). Sequences were quality-controlled using SeqScanner (v1.0), aligned against the EzBioCloud (18) database (https://www.ezbiocloud.net/identify), and assigned to the most similar reference strains. Isolates with sequence similarity below 98.65% (19) were considered unknown species.

### DNA extraction and purification

Tris-EDTA buffer (TE) and lysozyme were added to samples containing at least 1 mL of bacterial culture, followed by incubation at 37°C for 1 hour or overnight at room temperature until the suspension turned milky white. Genomic DNA extraction was performed using the Megan magnetic bead extraction kit, following the BGI automated procedure. After elution, 5 µL of RNase was added to the liquid, and the mixture was centrifuged for 5 min at 1,000 × *g* at 4°C and stored.

### Library generation and sequencing

DNA concentration was measured using the BR reagent. Qualified genomic DNA samples were randomly fragmented to construct double-ended libraries with fragment sizes of 300–400 bp. Sequencing was performed using the BGI DNBSEQ-T1&T5 platform, generating at least 4 GB of data per sample for subsequent analyses.

### Whole-genome assembly and analysis

Reads were filtered to a minimum length of 150 bp using Fastp (20) (v 0.20.1), followed by internal script-driven k-mer analysis to investigate the optimal genome size and heterogeneity. Clean data with a genome coverage of approximately 100× were selected for genome assembly and rRNA gene extraction, as described by Zou et al. (4). The CheckM (21) (v1.1.3) “lineage_wf” workflow was employed for genome quality assessment, considering genomes with completeness >90% and contamination <5% as high-quality genomes, and those with completeness >85% and contamination <5% as medium-quality genomes. Genomes with contamination rates > 5% were identified as representing multiple strains. The DAS Tool (22) (v1.1.2) was used, along with CONCOCT (23) (v1.1.0), MaxBin (24) (v2.2.7), and MetaBAT (25) (v2.15 with parameters --superspecific -m 2500), to aggregate results from the binning of genomes with contamination >5% to achieve separation of multi-strain genomes. Manual checks were conducted based on the G + C percentage and sequencing depth graphs for confirmation. In total, 548 high-quality genomes were obtained, termed the Yak Fecal Bacteria Genome Reference (YFR).

### Gene annotation, species annotation, and phylogenetic tree construction

Prokka (26) (v1.14.6) was used for gene and protein annotation of all assembled and isolated genomes (parameters --kingdom Bacteria --addgenes --addmrna --mincontiglen 200). Of these, 62.86% and 62.27% of the genes and proteins were classified as unknown, respectively. GTDB_Tk (27) (v2.1.0 with database release r207, “classify_wf” function, and default parameters) was used for species annotation and classification of strain genomes. A total of 332 genomes in the YFR were successfully annotated into 43 known species. For 216 genomes in the YFR annotated as unknown species, dRep (28) (v.2.5.4; parameters: --length 50000 --MASH_sketch 10,000 P_ani 0.90 S_ani 0.95 --cov_thresh 0.3) was used to cluster them into 29 non-redundant species-level clusters with a 95% ANI cutoff value. Phylophlan (29) (v3.0.60 with parameters --diversity high --fast --verbose) was applied for constructing a phylogenetic tree of 548 high-quality genomes in the YFR, followed by visualization using iTOL (30) (v6.8.1).

### Abundance of the YFR strains in metagenomic samples

A total of 388 yak metagenomic sample sequencing reads were downloaded from the CNGB Sequence Archive (CNSA) (https://db.cngb.org/cnsa/) of the China National GeneBank Database (CNGBdb) using the accession code CNP0001390. Using dRep (v.2.5.4), 77 species-level clusters of YFR were identified and compared with a collection called “Yakmeta,” composed of 4,991 species genome bins (SGBs) assembled from metagenomic data. Clustering was performed based on 95% average nucleotide identity (ANI >95%), resulting in 10 clusters showing overlap between the two sets. Bowtie 2 (31) (v2.4.2) was employed with the parameters “--end-to-end --sensitive -k 2” to align reads from the 388 samples with the union of the two sets (5,058 genomes). Unique alignments, aligning at both ends to the same overlapping group, with an identity of at least 0.95, were retained. In addition, 100 paired-end reads were used to support the presence of a given species in each sample (following the filtering criteria outlined by Li et al. (14)), and their relative abundance was calculated using the same formula as that used by Qin et al. (32).

### Comparison with the Hungate1000 collection and manually collected cultured strains

A total of 410 rumen genomes from the Hungate1000 project (33) (https://genome.jgi.doe.gov/portal/TheHunmicrobiome/TheHunmicrobiome.info.html) and 107 manually collected rumen isolate genomes (defined as “Other”) were downloaded (Table S3) (34–37). These were combined with 548 high-quality genomes from the YFR. The dRep tool (v.2.5.4; parameters: --length 50000 --MASH_sketch 10,000 P_ani 0.90 S_ani 0.95 --cov_thresh 0.3) was used to cluster genomes with a 95% ANI cutoff, resulting in 417 non-redundant species-level clusters. Phylophlan (v3.0.60) was used to construct a phylogenetic tree, followed by visualization using iTOL (v6.8.1).

### Pan-genome analysis

Roary (38) (v3.13.0) was used for pan-genome analysis of the *Streptococcus cluster 53* (parameters: -e -n -v -s -i 95) and differential gene querying between isolate groups (other sources and the YFR). Core genes were defined as genes present in 99%–100% of the strains, soft core genes as those present in 95%–99% of the strains, shell genes as those present in 15%–95% of the strains, and cloud genes as those present in 0%–15% of the strains. In addition, genes present exclusively in one population and found in more than 50% of the samples within that population were identified as unique genes.

### BGC annotation

We used AntiSMASH (39) (v6.0.0) to predict biosynthetic gene clusters (BGCs) for secondary metabolites in the 548 high-quality genomes from the YFR. The specified parameters included “--cb-general --cb-knownclusters --cb-subclusters --asf --pfam2go --smcog-trees --genefinding-tool prodigal.” We used BiG-SCAPE (40) (v1.1.5) to cluster Gene Cluster Families (GCFs), yielding a total of 484 GCFs with the parameters “--mix --cutoffs 0.3 --mode auto,” and 86 Gene Cluster Clans (GCCs) with the parameters “--cutoffs 0.7.

A comparative analysis of *Paenibacillus* was conducted using the same methods and tool versions as those used for strain BGC annotation, involving a total of 27 genomes. Among them, 12 strain genomes originating from the YFR were clustered into five clusters, and 15 genomes were downloaded from NCBI. Of these 15 genomes, seven were selected from the GTDB annotation’s “fastani_reference” column, representing evolutionarily close genomes, while eight genomes were manually collected as representative genomes of extensively studied species within *Paenibacillus*. CORASON (40) (CORe Analysis of Syntenic Orthologs to Priority Natural Product Biosynthetic Gene Clusters) was used to visualize the phylogenetic and evolutionary relationships among strains sharing the same type of gene clusters.

### Annotation of carbohydrate-active enzymes

dbCAN (41) (v3.0) was used to annotate carbohydrate-active enzymes (CAZymes) and carbohydrate-active gene clusters (CGCs) in the YFR. The parameters used were “-c cluster -sp signalp-5.0 -g all -c CLUSTER --cgc_substrate --pul PUL.” We selected CAZymes that were aligned by at least two of the three tools, HMMER, dbCAN_sub, and DIAMOND, as the final results. We manually selected CGCs with all modules aligning to validated PUL sites in dbCAN-PUL and a similarity exceeding 60% as high-confidence CGCs.

### Carbon source utilization assays for bacterial species

In all, 50 strains, representing 50 easily cultivable aerobic bacterial species across 3 phyla, were assessed using a custom-designed carbohydrate array. This array was developed in accordance with the methodology outlined by Martens et al. (42), albeit with certain modifications to the original protocol. Flat-bottom 96-well plates (Costar) were utilized in this study. Each well was supplemented with 100 µL of a 2 × concentrated solution for each sterile carbohydrate stock—including arabinogalactan, cellulose, fructan, pectin, starch, and xylan—individually prepared in Milli-Q water. Furthermore, a positive control, consisting of the optimal medium for strain growth, and a negative control, comprising a medium devoid of carbohydrates, were established. Each culture was grown in its medium until the cell density reached 10^7^ CFU/mL. Then, the cultures were spun in a centrifuge at 4,000 rpm for 5 minutes. The resulting pellet was washed twice with PBS to remove any residual carbohydrates from the medium and then resuspended in 1 mL of 2 × concentrated carbohydrate-free medium. This 1 mL of culture was used to start 50 mL of the same medium at a 1:50 ratio. After that, 100 µL of this culture was added to each well with the carbohydrate solution in a 96-well plate, making the final volume 200 µL. Subsequently, the 96-well plate was positioned in a microplate reader, and the optical density (OD) at 600 nm (A600) was recorded at 2 hour intervals. The measurement for each strain was conducted for a minimum duration of 20 hours. The results indicated that the negative controls for all strains demonstrated a declining trend, whereas the positive controls adhered to the expected growth curve. To construct a heatmap incorporating relative growth values, data normalization was conducted in accordance with the method outlined by Desai et al. (43). Carbohydrate growth assays exhibiting an absorbance increase exceeding 0.1 were marked with an asterisk (*).

### Bacteriophage annotation

VirSorter2 (44) (v2.2.2) with the parameter “--min-length 500 --min-score 0.5 --prep-for-dramv” was used for the annotation of bacteriophages, while CheckV (45) (v1.0.1) was employed to assess the completeness and contamination of all bacteriophages, with the “end_to_end” program and “-d checkv-db-v1.5” options. A viral genome with 100% completeness was defined as a complete viral genome; those with completeness between 90% and 100% were categorized as high-quality genomes; and those with completeness between 50% and 90% were designated as medium-quality genomes. Complete and high-quality viral genomes were used for further clustering analyses. VConTACT2 (46) (v.0.11.34) was utilized for clustering amino acid sequences of Prodigal (47) (v2.6.3) annotated viral genomes, with parameters “--rel-mode 'Diamond' --pcs-mode MCL --vcs-mode ClusterONE --db 'ProkaryoticViralRefSeq88-Merged' --c1-bin ../cluster_one-1.0.jar”. Cytoscape (48) (v3.10.0) was used to display the clustering patterns of the viral genomes in comparison with the database.

RGI (49) (v 6.0.2) was used to query antibiotic resistance genes for all complete and high-quality viral genomes from the Comprehensive Antibiotic Resistance Database (CARD). Virulence factors were annotated by comparing gene sequences with VFDB 2022 using BlastP (50) (v2.11.0+) with an e-value of 0.01.

### Functional prediction of the viral genome in *Streptococcus cluster 53* and differential analysis of the host

From the 39 bacterial genomes belonging to the *Streptococcus cluster 53*, all viral genome sequences predicted by vcontact2 were deleted, and the remaining reads were marked as host sequences. Prokka (26) (v1.14.6) was used to predict the number of genes in both the host and viral genomes. Emapper (51) (v2.1.12) was used to predict KO, KEGG Pathways, and corresponding CAZymes for the genes. RGI (49) (v 6.0.2) was used to query antibiotic resistance genes for all viral genomes from the Comprehensive Antibiotic Resistance Database (CARD). Virulence factors were annotated by comparing gene sequences with the VFDB 2022 using BlastP (50) (v2.11.0+) with an e-value of 0.01. Fisher’s exact test was performed using R to determine the enrichment direction of the viral genomes and host strains.

## RESULTS

### Reference genomes of cultivated bacteria from the gut microbiota of the Qinghai-Tibet Plateau yak

A total of 1,192 bacterial isolates were obtained from 16 fresh yak fecal samples collected from the Qinghai-Tibet Plateau using aerobic and anaerobic culture conditions and four different growth media. A total of 988 strains were identified based on the 16S rRNA gene sequence, with preliminary annotation using the EZBioCloud database revealing representation across four phyla, 30 genera, and 67 species. In all, 50 bacterial isolates were identified only at the genus level, indicating potentially novel bacterial species (Fig. S2a). In total, 48 species (64.86%) were isolated using aerobic culturing, 17 species were exclusively isolated using anaerobic culturing, and 9 species were isolated under both conditions (Fig. S2b). We observed clear media preferences for the isolation of the different strains, with LB medium contributing the highest percentage (43.52%) of bacterial isolates (Fig. S2c).

**Fig 1:**
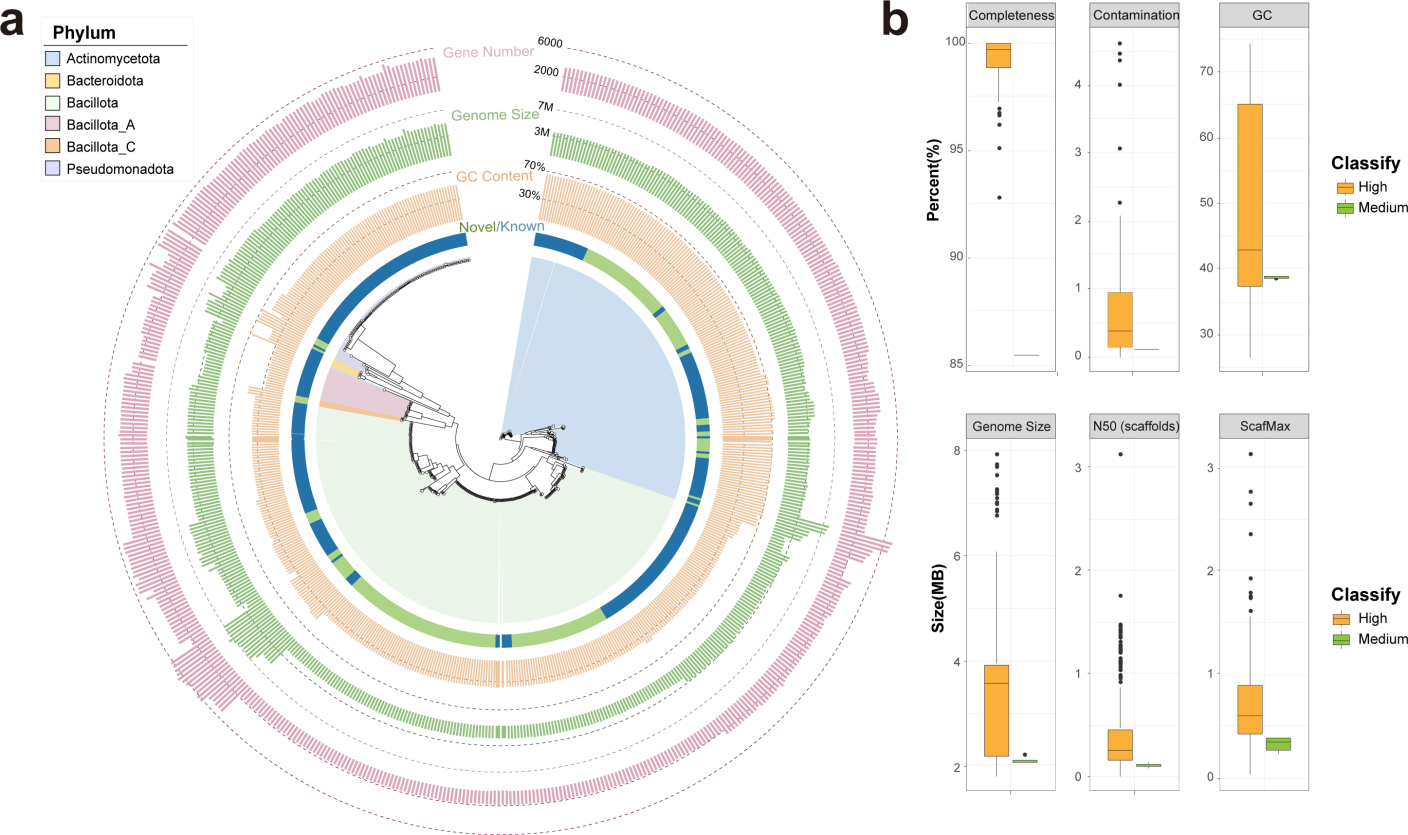
Taxonomic profile of 548 high-quality genomes in the YFR. (**a**) A phylogenetic tree was constructed based on 548 high-quality genomes, which were annotated into six phyla. Different colors in the innermost circle represent the novelty of the strains, where green indicates novel strains and blue indicates strains classified as known species. The outer circles display information on the GC content, genome size, and gene number. (**b**) Distribution of completeness, contamination, GC content, genome size, Scaffold N50, and max scaffold length for the 548 high-quality genomes and 6 medium-quality genomes in the YFR. The green box represents the medium-quality strain genome, and the orange box represents the high quality.

Subsequently, 526 re-streaked isolates providing broad coverage of the phylogenetic tree were selected for whole-genome sequencing and genome assembly. K-mer analysis revealed that 491 isolates represented a single strain, whereas 29 isolates exhibited chimerism involving two or three strains. To resolve these mixtures, metagenomic binning methods were employed, resulting in the identification of an additional 57 bacterial genomes. In summary, we obtained a collection of 548 high-quality genomes (completeness >90%, contamination <5%) and six medium-quality genomes (completeness >85%, contamination <5%) from fecal samples of yak, constituting the reference genome collection 'YFR'. The YFR data set encompasses genome sizes ranging from less than 2 Mb to a maximum of 7.9 Mb, with ScafN50 predominantly falling within the range of 150–450 kb. The GC content exhibited considerable variation, spanning from 26.6% to 74.4% (Fig. 1b). The GTDB database was employed for species annotation, revealing that 332 strains could be annotated to six phyla, 36 genera, and 51 known species. In addition, 216 strains were annotated only at the genus level, with no annotation information at the species level, indicating that these strains represent potentially novel species (Table S4). Hence, we subsequently clustered the 216 strains into 29 species-level clusters based on the average nucleotide identity (ANI) based on the 95% ANI criterion (Fig. 1a). The 29 species clusters were distributed among 6 phyla and 14 genera. Significant contributions to cultivated microbe reference genomes were made by members of phyla such as Bacillota (275, 50.19%), Actinomycetota (161, 29.38%), and Pseudomonadota (88, 16.06%). At the genus level, YFR comprised a large number of isolates belonging to *Arthrobacter*, *Escherichia*, *Streptococcus*, *Pradoella,* and *Enterococcus_B*. Notably, strains of *Streptococcus vicugnae* accounted for over 50% of the newly identified isolates.

### Augmenting cultural diversity of the ruminant gut microbiota

To investigate the representativeness of strains in the YFR, we examined the range of relative abundances of 77 species-level clusters previously reported for 388 yak gut microbiomes within the YFR (Methods). The results revealed that 66 species were represented in the metagenomic samples, with nearly all species (except *Paenibacillus cluster 16*, *Enterococcus_B lactis*, *Paenibacillus cluster 11*, *Bacillus pumilus*, and *Collinsella sp015062655*) having relative abundances exceeding 10^−3^ (Fig. S3). Thus, 33 species in the YFR data set encompassing 408 strains (including 190 potentially novel strains) represented abundant species (present in >50 metagenomic samples). In addition, 59 species comprising 489 strains (including 209 potentially novel strains) were detected in at least two metagenomic samples, and 18 species comprising 59 strains (eight potentially novel strains) were rare species (present in only one metagenomic sample). This implies that the strains in the YFR partially represent the microbial community of the yak gut, while also hosting a number of rare or low-abundance species.

**Fig 2:**
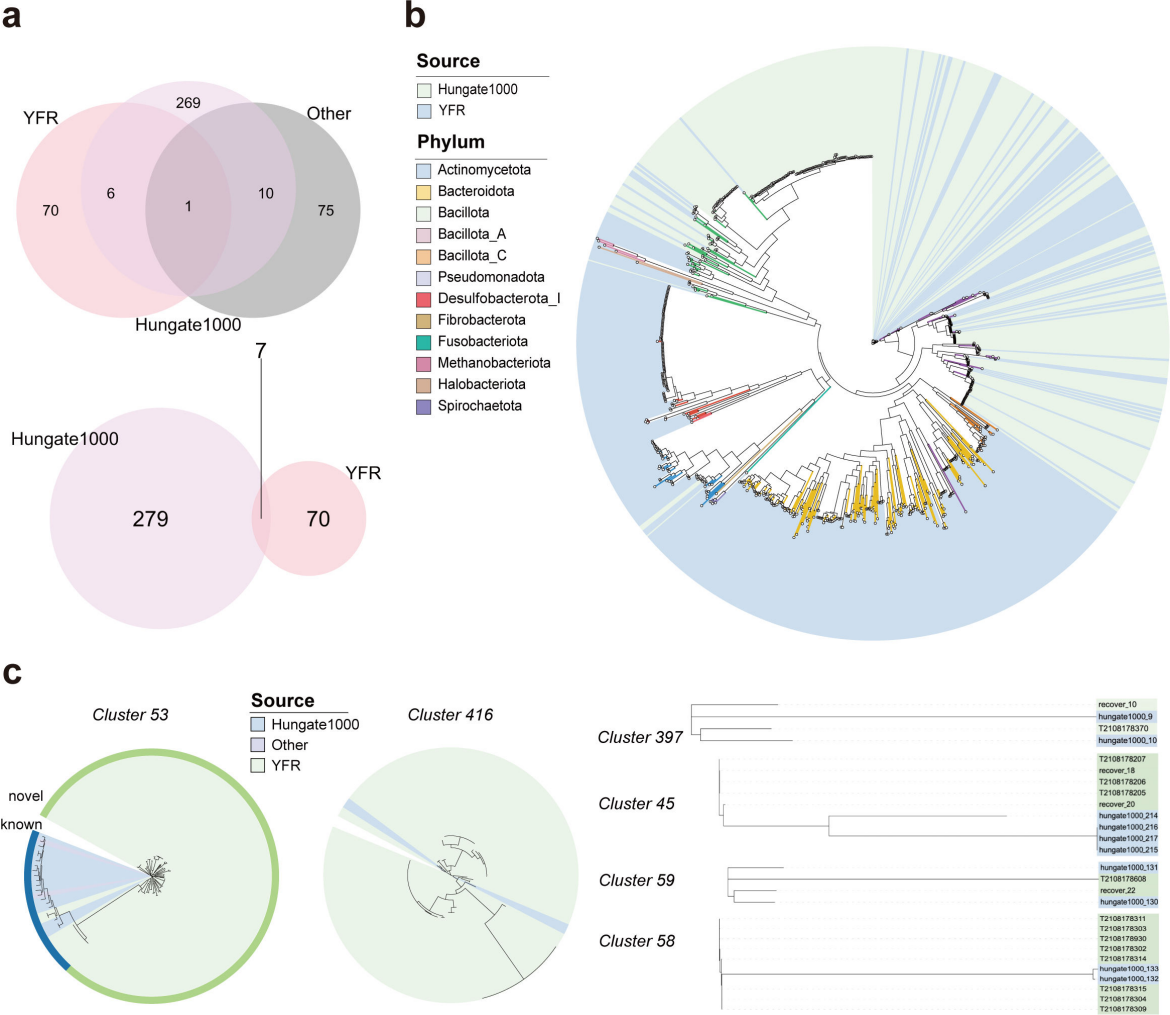
Comparison of genome information between the YFR and related databases. (**a**) Overlap and uniqueness among sets at the species level. (**b**) The phylogenetic tree was jointly constructed by Hungate1000 and the YFR, where the background represents different sources and branch colors indicate classifications at the phylum level. (**c**) The evolutionary distance between isolates with ANI differences < 5%, with the outer circle in *cluster 53* representing the novelty of the strains, where green indicates novelty.

To investigate the diversity of cultivated strains of YFR, we compared 410 genomes, encompassing 286 species from the Hungate1000 project and 107 manually collected rumen isolates. As expected, our database demonstrated significant novelty, with only seven species-level clusters overlapping with the existing strain collections (Fig. 2a; Table S5). In the YFR, 70 clusters (331 genomes) were not included in the published culturable bacterial database of ruminants. Our work increased the number of genomes of the cultivable strains of Pseudomonadota, Bacillota, and Actinomycetota from the ruminant gastrointestinal tract by 3.38-fold, 3.71-fold, and 5.03-fold, respectively, while also adding 20 new genera (243 strains) to these phyla. We envision that the genomic resources of YFR will serve as valuable references for investigating the functionality of the ruminant microbiota, elucidating host-microbiota interactions, and exploring the associations between environmental factors and microbiota composition.

For the overlapping species-level clusters, we visualized the evolutionary distances among these isolates from the YFR and published databases, observing significant genetic divergence between strains originating from different sources (Fig. 2b and c). To delve deeper into the functional differences between strains from different sources, we conducted a pan-genomic analysis of the *Streptococcus cluster 53* (143 strains, 124 isolated in the YFR, 19 published in other studies [13, 52, 53]) to determine which species exhibited the highest strain diversity among all overlapping clusters. Our analysis identified 1,292 core genes (at least existing in 141 strains), accounting for 24.66% of all genes in this cluster. In addition, soft core genes (existing in 135 strains), shell genes (existing in at least 21 strains), and cloud genes (existing in at least one strain) were identified and included 66, 620, and 3,261 genes, representing 1.26%, 11.83%, and 62.24% of the total genes, respectively (Fig. S4a). We found that strains from different sources possessed numerous source-specific genes, which may represent unique functions adapted to the host environment. Unique core genes in each source were defined as genes exclusively present in one source and found in more than 50% of the samples within that population. We identified 120 unique genes in the YFR strains and 51 unique genes in the strains from other published studies. We surveyed the functions of the unique core genes and found that the YFR strains exhibited an enrichment of genes potentially associated with high-altitude adaptation, envisioning a cycle where excreted bacteria recolonize the yak after being exposed to the harsh environment. These genes include *ktr*A, which encodes the Ktr system potassium uptake protein A, suggested to contribute to adaptation to high altitudes and high salinity (54); *pdg1*, which encodes ultraviolet N-glycosylase/AP lyase involved in repairing DNA damage caused by high-intensity UV radiation; and *oxyR*, which encodes a hydrogen peroxide-inducible gene activator (55) likely engaged in combating oxidative stress and adapting to low-oxygen tension environments. Conversely, YFR lacks genes related to multidrug resistance, such as the gene encoding the putative multidrug export ATP-binding/permease protein and putative multidrug resistance protein *MdtD*. This absence may be attributed to less pollution from human activities on the Qinghai-Tibet Plateau, resulting in yaks carrying fewer antibiotic resistance genes (54) (Fig. S4b).

### YFR comprises bacteria with the potential to synthesize novel natural products

To explore the potential for the production of secondary metabolites in the isolated strains, we employed AntiSMASH, which led to the identification of 2,607 Biosynthetic Gene Clusters (BGCs) encoded within the 540 high-quality genomes. No BGCs were detected in the eight strains with genome sizes of less than 3.07 Mb. In the YFR collection, each genome harbored, on average, 4.83 BGCs. Among the predicted BGCs, 94.2% had lengths exceeding 10 kb, with 79.85% of the BGC regions not located on the contig edge, supporting a high confidence of prediction (56). The BGCs were classified into seven groups: nonribosomal peptide synthetases (NRPS, 342), ribosomally synthesized and post-translationally modified peptides (RiPPs, 850), terpenes (305), others (674), type I polyketide synthases (PKSI, 8), other PKSs (PKSother, 358), and PKS/NRPS hybrids (70). BGCs belonging to the RiPPs category were found in bacteria from six phyla, whereas the PKSI category was specifically observed in the Pseudomonadota and Actinomycetota phyla (Fig. S5).

**Fig 3:**
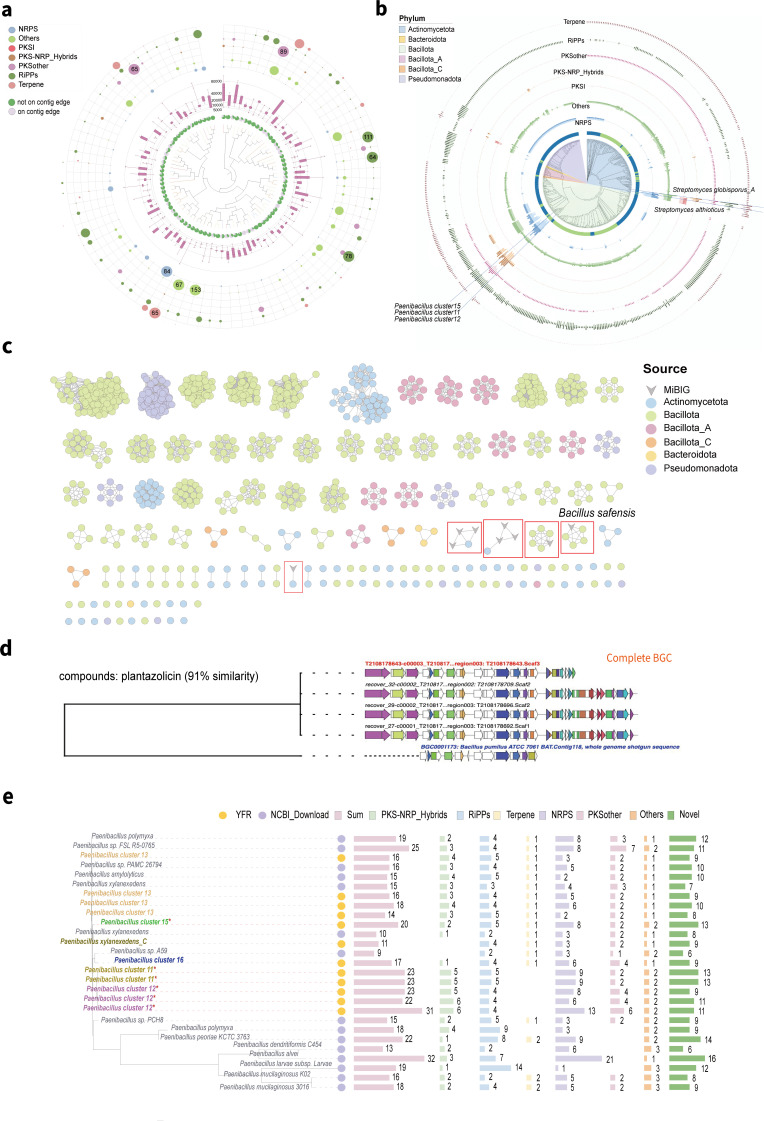
Distribution of BGCs in the YFR genomes. (**a**) Hierarchical clustering tree of 86 GCCs in the YFR, representing (from inner to outer) the proportion distribution of BGCs on contig edges, length distribution of BGCs, and distribution of BGC classes in GCCs. The colors of the bubbles indicate different BGC classes, and the size of the bubbles represents quantity. To enhance visibility, the maximum bubble size was set to 50, with BGC class bubbles larger than 50 labeled with numbers. (**b**) Distribution of seven BGC classes at the strain level in concentric circles, where darker columns represent the number of annotated BGCs and lighter columns represent the number of GCFs. Five species exhibiting a high potential for BGC synthesis are highlighted in the phylogenetic tree with blue lines. (**c**) Sequence similarity network of RiPPs. Edges drawn between nodes correspond to pairwise distances. Nodes represent sequences of BGC domains, with information about the phyla displayed in circles of different colors, and “v” shapes indicating BGCs from the MIBIG database. The red boxes highlight the YFR-annotated BGCs that cluster with the reference BGC. (**d**) The GCF clustered with a validated BGC to produce Plantazolicin. (**e**) Comparison of the number and types of BGCs produced by 12 *Paenibacillus* strains in the YFR with those closely related or widely studied *Paenibacillus* species in their evolutionary tree. Strains with gray font are downloaded strains, and strains with colored font are from the YFR, with different colors indicating different clusters. Species marked with an asterisk (*) are high-BGC-producing species in the *Paenibacillus* genus.

The BGCs were classified into 86 Gene Cluster Clans (GCCs) and 484 Gene Cluster Families (GCFs) (Fig. 3a). Among the GCFs, 326 contained at least one complete BGC. Only 25 GCFs matched validated BGC clusters in the Minimum Information about a Biosynthetic Gene cluster (MIBiG [57] release 3.1). The novel GCFs identified in this study may potentially encode previously uncharacterized secondary metabolites or unique variations of known metabolic pathways. The RiPPs category, with the highest BGC diversity and the widest range of hosts present in six phyla, was clustered into 151 GCFs, of which 84 GCFs included more than one BGC. The GCFs network of RiPPs did not yield any GCFs that encompassed BGCs from different phyla and exhibited phylum-specific GCFs. The genomes of the majority of Bacillota species harbored a rich array of RiPPs types, warranting further functional exploration (Fig. 3b and c). Only five GCFs had validated family members in the MIBiG database. The GCF (including five BGCs) from *Bacillus safensis*, which clustered with a validated BGC to produce Plantazolicin in the MIBiG database, exhibited 91% similarity. This suggests that our BGC has the potential to increase the diversity of ultra-narrow-spectrum antibiotics (Fig. 3d).

At the species level, we observed five species clusters (nine strains) exhibiting great potential for the synthesis of secondary metabolites (BGC > 20 and at least five classes of BGC, Fig. 3b). These five species clusters included two known species, *Streptomyces althioticus* and S*treptomyces globisporus_A*, as well as three unidentified species from the *Paenibacillus* genus, namely, *Paenibacillus cluster11, cluster12,* and *cluster15*. We focused on all isolated species within the *Paenibacillus* genus, which included one known species and five potentially novel species discovered in this study. Notably, all these strains exhibited a high abundance of BGCs (16–31 BGCs). These BGCs were classified into six major categories (PKS-NRP_Hybrids, RiPPs, Terpene, NRPS, PKSother, and Others), and these BGCs demonstrated huge potential for the production of various antibiotics, such as macrobrevin, polymyxin, zwittermicin A, and pellasoren—a compound renowned for its anti-tumor properties (Table S6). To compare the BGCs in the potentially novel strains from the YFR and well-known strains within the *Paenibacillus* genus, we conducted a comparative analysis by acquiring genomic data from *Paenibacillus* species that are either evolutionarily close or extensively studied. Our analysis revealed that each strain affiliated with species *clusters 11* and *clusters 12* in the YFR harbored more than 20 BGCs and a notable number of BGCs within the PKSother and PKS-NRP hybrid categories compared to their well-studied counterparts (Fig. 3e). Further clustering of the predicted 513 BGCs from these strains resulted in a total of 225 GCFs. These comprised NRPS (128 GCFs), RiPPs (72 GCFs), PKSother (50 GCFs), PKS-NRPS_Hybrids (32 GCFs), others (25 GCFs), terpenes (8 GCFs), and PKSI (2 GCFs). Notably, unannotated strains from YFR contained 48 unique GCFs, with four GCFs harboring at least one complete BGC. This suggests that these unannotated strains from the YFR may support the synthesis of many compounds with specific but as-yet-undiscovered functionalities. In addition, a strain of *Streptomyces globisporus_A*, which possesses a genome size of 7.9 Mb, warrants attention. This strain harbors 37 BGCs spanning six compound categories distributed across 37 GCFs. Notably, 13 BGCs from this strain clustered with experimentally validated BGCs in the MIBiG database, suggesting that this strain has great potential to synthesize a variety of compounds, including radamycin/globimycin, thioholgamide A/thioholgamide B, coelibactin, frontalamide B, and C-1027 (Fig. S6). In conclusion, the YFR constitutes a valuable resource for exploring novel secondary metabolites and advancing the discovery and characterization of natural products.

### Exploring the potential of yak gut bacteria for the digestion of complex polysaccharides

**Fig 4:**
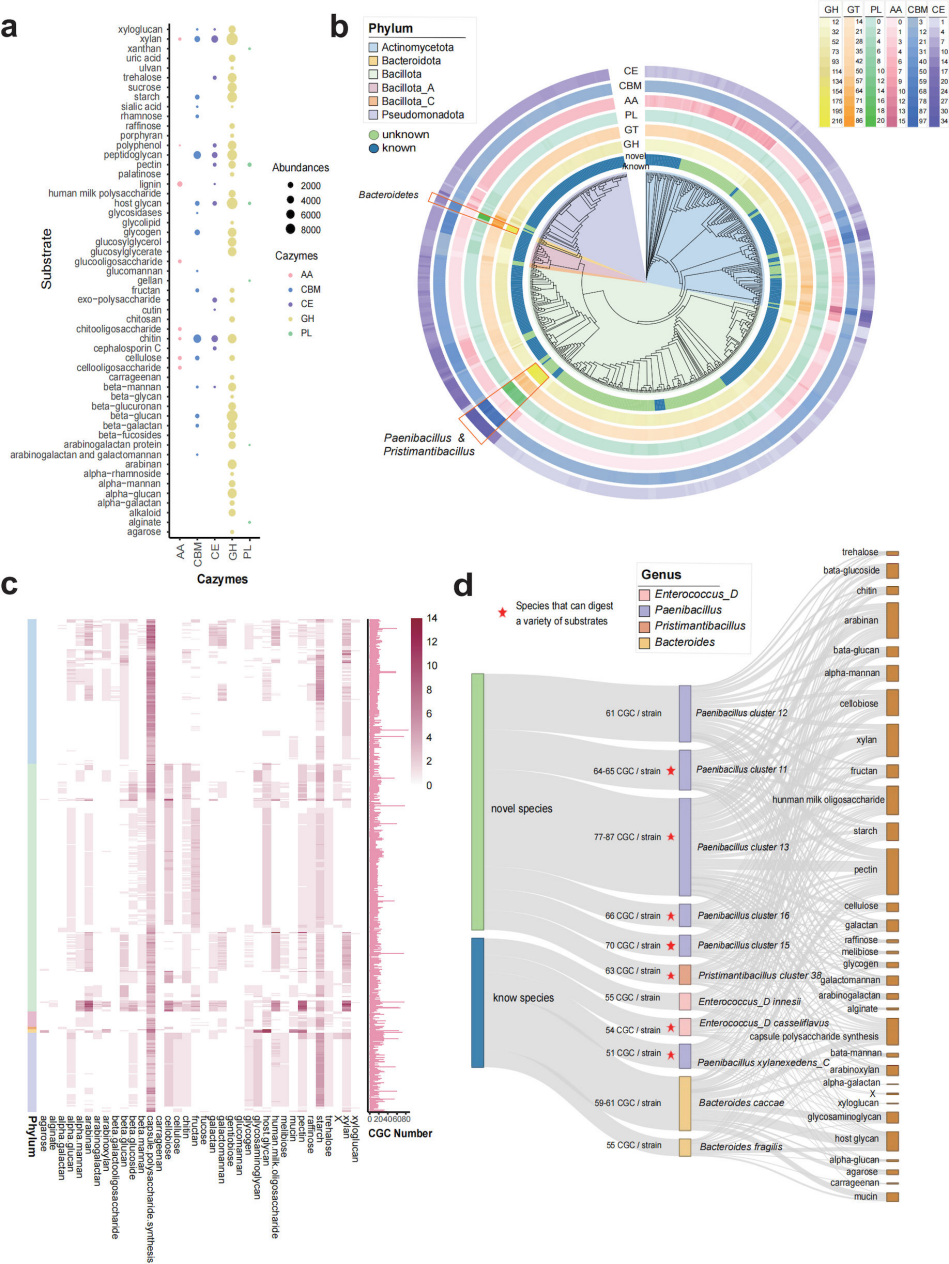
Abundant CAZymes and CGCs in the YFR genomes. (**a**) Correspondence between CAZymes and the types of digested substrates, where bubble colors represent different categories of CAZymes, and bubble sizes indicate the number of CAZymes corresponding to the digestion of that substrate. (**b**) Distribution of CAZymes at the strain level, with the second circle representing the novelty of strains. The heatmap rings of different colors represent the number of annotated CAZymes in each genome for different categories, with the color indicating the numerical distribution range, as shown in the legend. The highlighted area in the red boxes represents strains with abundant CAZymes production potential. (**c**) Distribution of the number of CGCs in the strains and their correspondence with the digested substrates. (**d**) Correspondence between 11 species containing multiple CGCs (>50) and digested substrates.

We examined the potential for the synthesis of carbohydrate-active enzymes in the YFR genomes using dbcan3 to annotate CAZymes and selected strains harboring CAZymes annotated by more than two methods (10). Using this approach, 61,679 CAZymes were annotated in our collection. The most abundant CAZyme classes were GH (45.66%) and GT (29.5%). The predicted CAZymes were involved in the digestion of 53 substrates, with chitin, peptidoglycan, and xylan being the primary substrates (Fig. 4a). The CAZyme families associated with cellulose degradation mainly include AA3, CBM63, GH74, CBM2, CBM9, GH6, CBM16, CBM6, AA10, GH48, CBM3, and CBM46. These families were predominantly found in *Paenibacillus*, *Streptomyces*, and *Cellulomonas* (Fig. S7).

Although multiple studies have indicated that cellulose-degrading bacteria in the rumen mainly belong to Bacteroidota, our analyses revealed that aerobic bacteria from the gut belonging to the *genera Paenibacillus and Pristimantibacillus* also possess significant potential for the synthesis of CAZymes (Fig. 4b). The relaxed culture requirements of these bacteria render them highly suitable for large-scale cultivation and industrial applications.

To further explore the ability of these strains to digest complex polysaccharides, we analyzed the distribution of CAZyme gene clusters (CGCs) in their genomes. CGCs, defined by Zheng et al. (58), contain CAZymes and at least one characteristic gene in potential Polysaccharide Utilization Loci (PUL) that may play vital roles in microbial communities by aiding in carbohydrate decomposition and utilization. In our 548 genomes, 11,224 CGCs were predicted, which enabled the digestion of 35 known substrates. We found that 30.98% of all CGCs were rich in capsule polysaccharide synthesis and starch digestion (Fig. 4c). Our results also indicated that strains from the Bacillota, Bacillota_C, and Pseudomonadota phyla in the YFR harbored numerous CGCs related to cellulose and hemicellulose degradation, respectively. However, these computationally predicted CGCs have not been experimentally validated because of a lack of isolates for characterization. The addition of YFR strains broadens the range of strains available for future experimental validation of CGCs.

We examined 11 species in the YFR containing multiple CGCs (>50) present in four different genera: *Bacteroides, Enterococcus_*D*, Pristimantibacillus*, and *Paenibacillus*. Among them, six species are potentially novel. We identified seven species (including *Enterococcus*_D, *Enterococcus casseliflavus*, *Paenibacillus xylanexedens*_C, and five potentially novel species from *Paenibacillus*) with the ability to digest a variety of substrates (>20), such as pectin, xylan, fructan, galactan, galactomannan, and arabinan (Fig. 4d). Comparing these CGCs with PUL characteristics, apart from strains from the Bacteroidota phylum, strains from the Bacillota and Actinomycetota phyla harbored PULs in the validated PUL database. This indicates a huge untapped potential for the digestion of complex polysaccharides by members of the Bacillota and Actinomycetota phyla.

We further validated the utilization of the predominant polysaccharides in plants by the isolated microbial species through single-carbon-source experiments, aiming to clarify whether each strain can target one or multiple specific polysaccharides. Our results (Fig. S8; Table S7) revealed significant variations in carbon source preferences among the 50 microbial species tested on six polysaccharides. Xylan and fructan were widely utilized by diverse microbial species, particularly those from the Bacillota and Actinomycetota phyla, demonstrating their efficient degradation capabilities for metabolizable polysaccharides. By contrast, utilization of cellulose was limited, with only one strain exhibiting detectable utilization activity. Notably, multiple screened strains displayed the ability to degrade complex polysaccharides arabinogalactan and pectin, suggesting their potential as valuable candidates for further microbial and enzymatic development.

### Bacteriophage in the YFR

**Fig 5:**
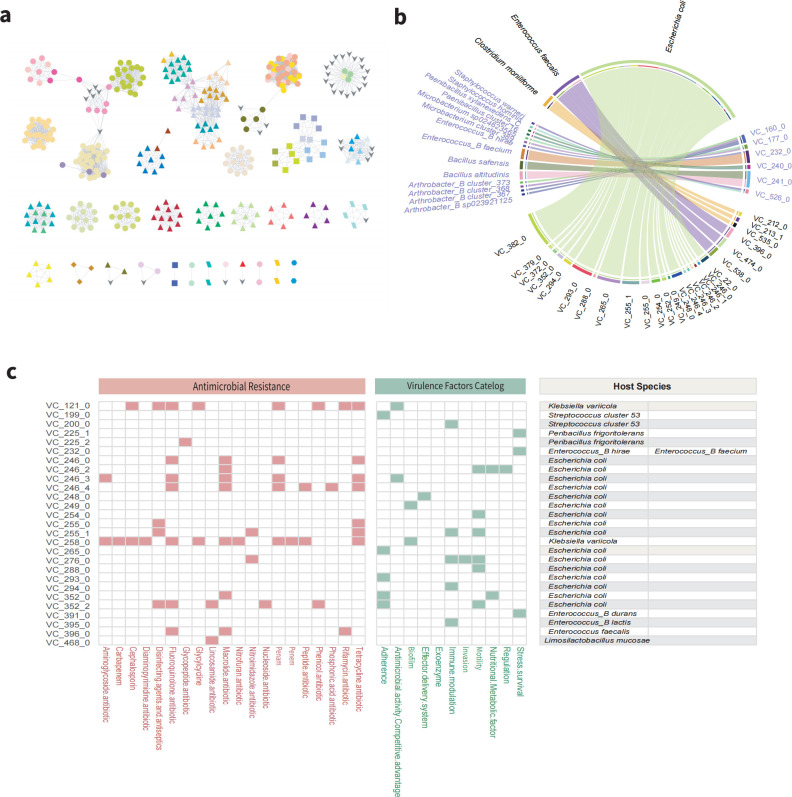
Summary of phages in the YFR genomes. (**a**) Gene-sharing network of phages in the YFR, where nodes represent phages. Different colors represent different virus clusters, and different shapes represent different phyla, with parallelograms representing Actinomycetota, triangles representing Bacillota, squares representing Bacillota_A, diamonds representing Bacillota_C, hexagons representing Bacteroidota, ellipses representing Pseudomonadota, and V-shapes representing known phages in the current databases. (**b**) Distribution of VCs in bacterial species, showing bacterial species with multiple VCs categories and VCs with relatively broad host ranges. (**c**) VCs carrying ARGs and VFs along with their host sources.

To address the issue of inadequate isolation of phages from culturable bacterial strains in ruminal animals (59), we used VirSorter2 analyses. These analyses led to the prediction of 2,224 viral genomes in 323 strains of YFR, including 1,842 dsDNA phages and 382 ssDNA viral genomes. We were unable to predict the viral genomes of the remaining 225 strains. CheckV was used to assess the integrity and contamination of all phages. Of these, 80 were identified as complete phages, 96 as high-quality phages, and 283 as medium-quality phages. The median size of the viral genomes was 71,806 bp (interquartile range, IQR = 45,068-112,169 bp), with two phages exceeding 200 kb (Jumbo phages) (Fig. S9). *Escherichia coli* from the Pseudomonadota phylum was the main host for these bacteriophages, followed by *Streptococcus equinus*, *Enterococcus faecalis*, and *Enterococcus_B durans* (belonging to the Bacillota phylum). We clustered 459 medium-to-high-quality and complete viral genomes into 67 virus clusters (VCs), including 55 multiphage clusters and 12 singletons (Fig. 5A). Only nine clusters comprising 24 phages were annotated as known phages, which mainly belonged to the *Siphoviridae, Myoviridae,* and *Podoviridae* families, suggesting that phages from the bacteria of the YFR represent a novel and unexplored viral resource (Table S8).

We investigated the distribution of phages exceeding medium quality among hosts to determine whether they were biased toward specific bacterial taxa. We found that strains from the Pseudomonadota phylum in the YFR harbored up to six phages (IQR = 2-3), while genomes of strains from other phyla at most carried 3–4 phages (IQR = 1–2, Fig. S10). This distribution may be related to the host genome size, which has been reported to exhibit a weak positive correlation with the number of phages (60). We examined the relationship between VCs diversity and hosts, revealing that three bacterial species, *Clostridium moniliforme, Enterococcus faecalis,* and *E. coli*, carried multiple different VCs (≥3), suggesting potential genomic diversity, environmental adaptability, and complex ecological interactions with phages. In addition, six VCs (VC_160_0, VC_177_0, VC_232_0, VC_240_0, VC_241_0, and VC_526_0) showed a relatively broad host range, existing in multiple species without crossing genus boundaries (Fig. 5b), implying common susceptibility or interaction patterns with specific bacterial species.

Antibiotic resistance genes (ARGs) and virulence factors (VFs) were also assessed in all bacteriophages. The results showed that 14 of the 67 viral clusters carried ARGs. The ARGs carried by these viral clusters were mainly associated with fluoroquinolone, macrolide, tetracycline, and penam antibiotics. In addition, 21 viral clusters carried VFs, which were primarily related to motility, adherence, and immune modulation. *E. coli* was the host for most of these viral clusters (Fig. 5c). Interestingly, the VCs associated with *Klebsiella varricola* and *E. coli* were the sole carriers of both ARGs and VFs, whereas phages from other hosts harbored either ARGs or VFs. Given the potential role of phages in the transmission of features related to bacterial pathogenicity and antibiotic resistance, we speculate that this phenomenon may be associated with *Klebsiella varricola* and *E. coli* acting as opportunistic pathogens.

### Genetic and functional diversity of viral sequences in the genomes of isolated bacteria

**Fig 6:**
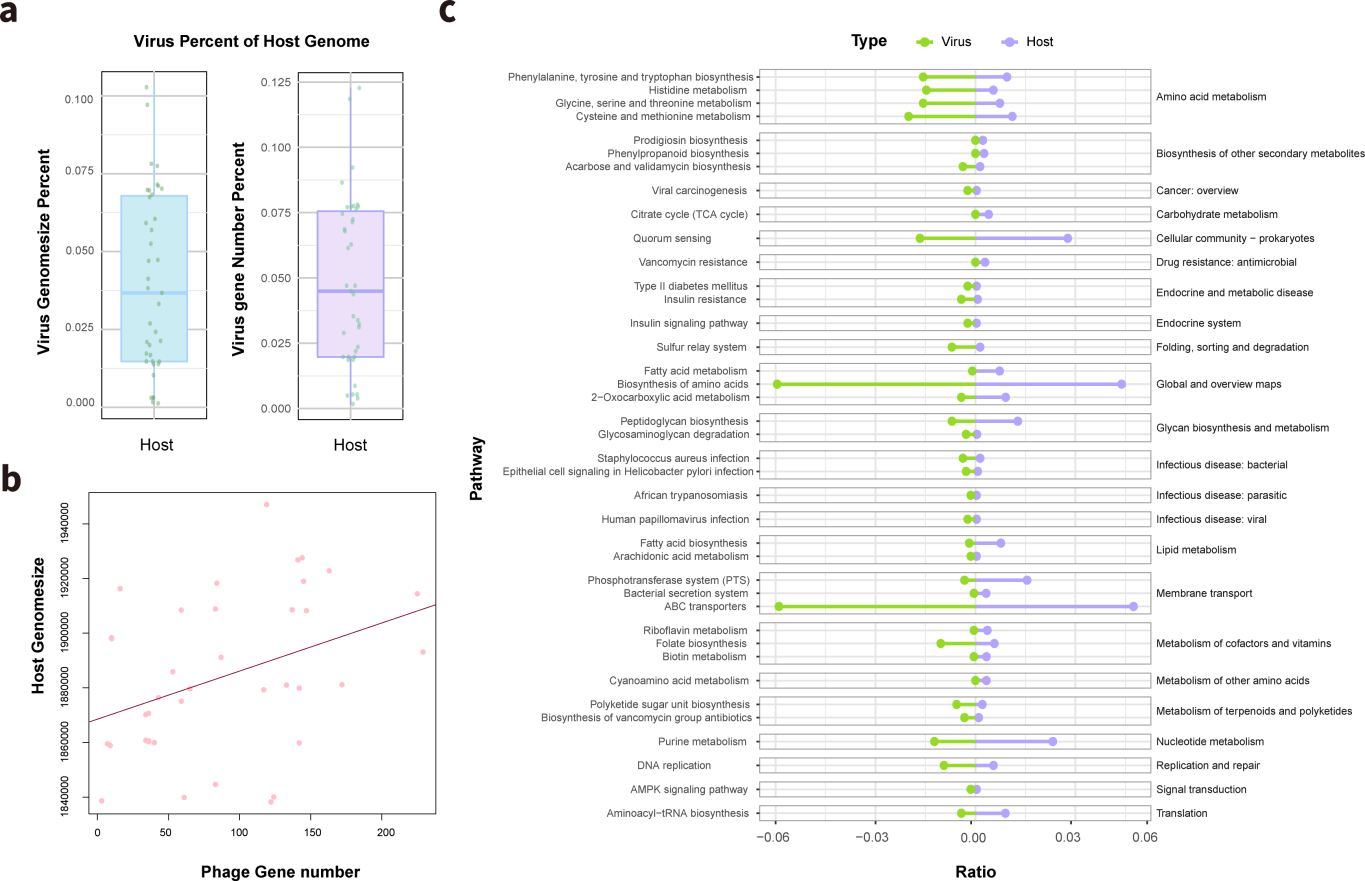
The viral genome enhances the phylogenetic diversity of the host. (**a**) Percentage of viral genome size and viral gene number in *Streptococcus cluster 53* strains. (**b**) Correlation between phage gene number and host genome size. (**c**) KEGG pathways enriched in the viral genome and host.

Cultured single bacterial genomes provide the potential for studying the interrelationship between the virus and the host of the bacterial species. We investigated the viral genome carried by different strains in relation to a novel isolated bacterial species (*Streptococcus cluster 53*). As a result, 72 viral genomes were predicted from the 39 cultured strains, including 57 incomplete viral genomes and 15 intact proviruses. The DeePhage tool was used to predict the lifestyle of the viruses, distinguishing between virulent and temperate types. Eight strains harbored 11 virulent viruses. The size of the viral genomes carried by all strains ranged from 527 to 194,602 bp, accounting for 0.13% to 10.28% of the host genome (Fig. 6a). A total of 3,492 genes were predicted in the viral genomes, and the number of genes in these viral genomes represented approximately 0.17%–12.27% of the total number of genes in the host genome (Fig. 6a). Furthermore, we observed significant correlations between the sizes of the viral and host genomes and the gene counts within these genomes (Fig. 6b).

Functional annotation was performed for the 3,492 genes in the viral genomes, among which 291 were identified as virulence factor (VF) genes, while no antibiotic resistance genes (ARGs) were annotated. VF genes are involved in immune modulation (51), adherence (222), and exoenzyme production (18). A total of 56 genes were predicted to be CAZymes involved in complex carbohydrate assembly and breakdown.

In addition, we investigated how the fusion of viral genome genes with host bacteria can enhance or strengthen their functions. We found that 90.9% of the genes in the viral genome regions are core genes that exist in over 32 strains of *Streptococcus cluster 53*, implying that these genes are crucial for the host bacterial species. Furthermore, we explored the differences in the enrichment of metabolic pathways between genes in the viral and host genomes. Our results show that genes in the viral genomes are primarily enriched in cationic antimicrobial peptide (CAMP) resistance, epithelial cell signaling in *Helicobacter pylori* infection, *Staphylococcus aureus* infection, ABC transporters, metabolism of terpenoids and polyketides, and folate biosynthesis, indicating that viral genes play important roles in these specific biological processes and metabolic pathways, potentially affecting the host’s antimicrobial resistance, infection processes, substance transport, and metabolic functions (Fig. 6c). Moreover, we observed that the functional disparities among strains were mitigated in the presence of viral genes, suggesting that viral influence confers supplementary functionalities, thereby enhancing functional similarity within strains of the same species (Fig. S11). For instance, the viral genomes of strains T2106134295 and T2108178176 encode key enzymes absent in the host strain, which facilitate folate synthesis and increase the diversity of ABC transporters (Fig. S12). The findings of our study highlight the valuable potential of cultured strains as a resource for further investigation into the intricate relationship between viruses and host strains.

## DISCUSSION

An increasing number of studies have recognized the critical role of the gut microbiota (61). At present, the investigation of cultivable strains and the development of high-quality reference genomes pertaining to the gastrointestinal microbiota of ruminants are confined to less than 15% of the isolates from the Hungate1000 project. In this study, we collected fecal samples from plateau yaks inhabiting high-altitude regions and subjected them to cultivation under eight different conditions. During the isolation of pure cultures from the yak gut microbiota, we observed that standard intestinal bacterial media optimized for human gut strains failed to support robust growth of yak-derived isolates, necessitating culture condition adjustments and optimization. This improved approach enabled us to obtain over 1,000 bacterial isolates and to produce 548 high-quality reference genomes of the intestinal microbes found in yaks. Among them, 39.42% (216) were annotated as being novel. The substantial new bacterial yield suggests the uniqueness of the microbiota within the intestinal tracts of animals inhabiting specialized environments, warranting further investigation.

Through systematic analysis of single bacterial genomes, we revealed the diversity of the high-altitude yak gut microbiota and provided insights into its functional potential. The observed functional repertoire, including the high prevalence of CAZymes for complex polysaccharide digestion, such as chitin and xylan, novel BGCs with potential for bioactive compound synthesis, and specific adaptations such as genes involved in UV damage repair (*pdg1*) and oxidative stress response (*oxyR*), likely reflect adaptations to the Qinghai-Tibet Plateau environment, encompassing responses to the herbivorous diet at high altitudes, low oxygen tension, intense UV radiation, and potentially unique local flora. While this geographic specificity provides valuable insights into microbial adaptation to extreme conditions and identifies potentially unique functional traits, it underscores that the exact composition and relative abundance of these functional elements may vary in yaks from different ecosystems. Furthermore, we successfully uncovered numerous BGCs, and exploration at the level of gene cluster families (GCFs) revealed that 94.83% of the BGCs were unexplored. This proportion closely resembles the unexplored secondary metabolite potential of 96.8% observed in marine prokaryotes (62), emphasizing the vast untapped biosynthetic potential within the yak gut microbiota. We also found that unknown strains of the *Paenibacillus* genus have significant potential for synthesizing antibiotics and anti-tumor substances. These findings imply that future research efforts should investigate the functional characteristics of these bacteria from harsh environments.

The investigation of microbial strains within the intestinal microbiota of ruminants, which are capable of efficiently degrading plant fibers such as cellulose and hemicellulose, has consistently attracted significant scholarly interest. Our study confirmed the presence of numerous enzymatically potent strains in the hindgut microbiota of ruminants that are primarily involved in polysaccharide degradation. Unlike the predominant microbial communities responsible for digestion in the rumen, some strains in the hindgut, such as those belonging to the *Paenibacillus* and *Pristimantibacillus* genera with lower culture requirements, may play a crucial role in polysaccharide degradation. Our research suggests that 11 high-yield CGC species from the *Bacteroides*, *Enterococcus_D*, *Pristimantibacillus*, and *Paenibacillus* genera may be broadly involved in the degradation of 20 potential substrates, delineating the potential efficiency of aerobic strains compared to anaerobic strains in polysaccharide degradation still requires further validation. Thus, future research should focus on the functional validation of the identified CGCs to gain a deeper understanding of their specific roles in complex carbohydrate degradation. However, detailed functional analyses are required to confirm this hypothesis. This could offer new insights and solutions in agriculture, feed production, and bioenergy.

While metagenomics offers extensive insights into the structure and function of microbial communities, the role of pure culture work remains indispensable. The isolation and characterization of single microbial strains are crucial for elucidating physiological traits, metabolic mechanisms, phage-host interactions, and specific biotechnological applications, such as antibiotic discovery and synthetic biology. Its significance remains irreplaceable in the era of omics. In our project, exploring bacteriophages not only offers clues to understanding the spread of antibiotic resistance and virulence factors in the gut microbiota of plateau yaks but also highlights the potential role of host-associated bacteriophages in this process. Our research on *Streptococcus cluster 53* revealed that viral genomes significantly influence the metabolic pathways and biological processes of host strains and contribute to the homogenization of functional characteristics across different strains of the same species. This finding underscores the crucial role of viral genomes in shaping the adaptability and functional diversity of host strains. Future investigations into viral genomes within pure culture strains will enhance our understanding of the relationship between viruses and host strains, providing novel perspectives and methodologies for developing innovative antibacterial strategies and microbial engineering applications.

### Limitations and future perspectives

While the YFR serves as a resource for investigating yak gut microbiota, it is important to acknowledge several limitations inherent in the current study. First, the restricted geographic sampling (*n* = 16 from a single plateau region) may not adequately represent the full diversity of yak gut microbiota, particularly for age- and location-specific variants. Second, the absence of temporal sampling across different seasons. In addition, while our cultured isolates show strong concordance with metagenomic data for dominant species (section Augmenting Cultural Diversity of the Ruminant Gut Microbiota), the inherent biases of culturomics approaches likely led to underrepresentation of fastidious or anaerobic microorganisms that are challenging to culture. These limitations may constrain the generalizability of our current findings to broader yak populations and ecosystems.

Future research should focus on several key directions: first, the implementation of comprehensive sampling strategies that encompass diverse geographic regions, altitudinal gradients, and seasonal variations to capture the full ecological range of yak microbiota. Second, the integration of advanced culturomics techniques with metagenomic and metatranscriptomic analyses is essential to overcome culturability biases and enable strain-level functional characterization. In addition, the use of gnotobiotic animal models is recommended to experimentally validate the physiological roles of isolated strains, particularly those encoding putative metabolic enzymes (e.g., cellulases, xylanases) relevant to high-fiber digestion. Such multi-dimensional approaches will significantly enhance our understanding of the yak-microbiome symbiosis and its adaptations to extreme plateau environments.

## Data Availability

The sequencing and genome data generated in this study were deposited in the CNGB Sequence Archive (CNSA) (63) of the China National GeneBank DataBase (CNGBdb) (64) and are accessible under accession number CNP0004162. In addition, all bacterial strains from the YFR have been stored in the China National GeneBank (CNGB), a non-profit organization in China dedicated to public services.

## SUPPLEMENTAL MATERIAL

Supplemental material msystems.00367-25-s0001.pdf Supplemental information and figures.

Supplemental tables msystems.00367-25-s0002.xlsx Tables S1 to S8.

ASM does not own the copyrights to Supplemental Material that may be linked to, or accessed through, an article. The authors have granted ASM a non-exclusive, world-wide license to publish the Supplemental Material files. Please contact the corresponding author directly for reuse.
