# Supplementary material for: Cultivation and sequencing of microbiota members unveil the functional potential of yak gut microbiota
Source: mSystems. 2025 Aug 15;10(9):e00367-25. doi: 10.1128/msystems.00367-25 (PMC12455938; doi:10.1128/msystems.00367-25)

Supplementary Information

The specific formulations of four different agar media:

a. Medium 1. Modified MPYG Anaerobic Medium:

The components of the solution per liter are as follows: Trypticase peptone (5.00 g), Peptone (3.00 g), Peptone from soya (2.00 g), Polypeptone (1.00 g), Yeast extract (10.00 g), Beef extract (5.00 g), Glucose (5.00 g), Tween 80 (0.50 ml), Maltose (0.50 g), Cellobiose (0.50 g), Soluble starch (0.50 g), Glycerol (0.50 ml), K_2HPO_4 (2.00 g), Cysteine-HCl x H_2O (0.50 g), Na_2S (0.25 g), Resazurin (1.00 mg), Salt solution (40.00 ml), Trace element solution (10.00 ml), Vitamin solution (10.00 ml), Haemin solution (10.00 ml), Vitamin K_1 solution (0.20 ml), Clarified rumen fluid (60 ml), Sheep blood (100 ml), and Agar (15 g).

The salt solution (DSMZ Salt solution) consists of the following components: $CaCl_2$ x 2 H_2O (0.25 g), $MgSO_4$ x 7 H_2O (0.50 g), K_2HPO_4(1.00 g), KH_2PO_4 (1.00 g), $NaHCO_3$ (10.00 g), NaCl (2.00 g), and distilled water (1000.00 ml).

The trace element solution (DSMZ Trace element solution) includes the following components: nitrilotriacetic acid (1.50 g), $MgSO_4$ x 7 H_2O (3.00 g), $MnSO_4$ x H_2O (0.50 g), NaCl (1.00 g), $FeSO_4$ x 7 H_2O (0.10 g), $CoSO_4$ x 7 H2O (0.18 g), $CaCl_2$ x 2 H_2O (0.10 g), $ZnSO_4$ x 7 H_2O (0.18 g), $CuSO_4$ x 5 H_2O (0.01 g), $KAl(SO_4)^2$ x 12 H^2O (0.02 g), H_3BO_3 (0.01 g), Na_2MoO_4 x 2 H_2O (0.01 g), $NiCl_2$ x 6 H_2O (0.03 g), Na_2SeO_3 x 5 H_2O (0.30 mg), and distilled water (1000.00 ml).

The vitamin solution consists of the following components: biotin (2.00 mg), folic acid (2.00 mg), pyridoxine-HCl (10.00 mg), thiamine-HCl x 2 H_2O (5.00 mg), riboflavin (5.00 mg), nicotinic acid (5.00 mg), D-Ca-pantothenate (5.00 mg), vitamin B_{12} (0.10 mg), p-aminobenzoic acid (5.00 mg), lipoic acid (5.00 mg), and distilled water (1000.00 ml).

This modified MPYG meidium supplemented with clarified rumen fluid and sheep blood.

b. Medium 2. Modified MRS Anaerobic medium:

The components of the solution per liter are as follows: peptone (10.0 g), beef paste powder (5.0 g), yeast powder (4.0 g), glucose (20.0 g), Tween 80 (1.0 ml), dimethyl hydrogen phosphate (2.0 g), sodium acetate (5.0 g), triammonium citrate (2.0 g), $MgSO_4.7H_2O$ (0.2 g), $MnSO_4 \cdot 4H_2O$ (0.05 g), cysteine-HCl x H_2O (0.50 g), Na_2S (0.25 g), sheep blood (100 ml), clarified rumen fluid (60 ml), and agar (15 g). The final pH is 6.2±0.2.

This modified MRS meidium supplemented with clarified 10% rumen fluid, 6% sheep blood and reducing substances Na_2S ,Cysteine-HCl x H_2O.

c. Medium 3. Modified BHI Anaerobic Medium:

The components of the modified BHI medium per liter are as follows: tryptone (10.0 g), sodium chloride (5.0 g), disodium phosphate (2.5 g), glucose (2.0 g), beef heart extract (500 ml), cysteine-HCl x H_2O (0.50 g), Na_2S (0.25 g), sheep blood (100 ml), clarified rumen fluid (60 ml), and agar (15 g). The final pH is 7.4±0.2.

This modified BHI medium is supplemented with clarified rumen fluid, sheep blood, and reducing substances Na_2S and cysteine-HCl x H_2O.

d．Medium 4．Modified LB Anaerobic Medium:

The components of the modified LB medium per liter are as follows: tryptone (10.0 g), yeast extract (5.0 g), NaCl (10.0 g), agar (15.0 g), cysteine-HCl x H_2O (0.50 g), Na_2S (0.25 g), sheep blood (100 ml), and clarified rumen fluid (60 ml). The final pH is 7.2.

This modified LB medium is supplemented with clarified rumen fluid, sheep blood, and reducing substances Na_2S and cysteine-HCl x H_2O.

The preparation of all aerobic culture media is based on that of anaerobic culture media with the reduction of the addition of Na_2S and cysteine-HCl x H_2O components.

Supplementary Figure Legends

Figure S1. The pipeline of isolated bacterial strains from Yak fecal samples.

Figure S2. The species distribution using different isolation conditions based on the taxonomic information by 16S rRNA genes. (a).Annotation of 988 isolated strains at different taxonomic levels. The graph displays only the top five most frequently observed taxa, with the remaining lineages categorized as "Others." The white bars indicate the proportion of novel strains within the species. (b).Venn diagram illustrating microbial species isolated under aerobic and anaerobic conditions. (c).Upset plot illustrating the shared and distinct taxon numbers across various cultivation environments.

Figure S3. The relative abundance of 77 representative species clusters in 388 Yak fecal samples. The abundance range and occurrence across 388 samples for 77, species in the YFR, different colors represent different phyla. Species marked with an asterisk (*) are novel species in the YFR.

Figure S4. Genome information of *Streptococcus cluster 53.* (a).The evolutionary tree of *Streptococcus cluster 53* from diverse sources (YFR, Hungate1000 or Other) alongside a pan-genome matrix, depicting the presence and absence of core and

accessory genes. (b).The distribution of unique core genes in each source. Distribution heatmap of unique genes in *Streptococcus Cluster 53* from different sources. The upper-left section represents genes exclusively present in *Streptococcus Cluster 53* from the YFR, while the lower-right section indicates genes present in *Streptococcus Cluster 53* from both Hungate1000 and other collections.

Figure S5. Distribution and proportion of BGC classes across different phyla. The stacked bar chart illustrates the distribution and proportion of BGC classes across different phyla, while the pie chart shows the percentage of each BGC class among all identified BGC.

Figure S6. The BGCs in *Streptomyces globisporus A*. (a). Each shape represents a BGC (Biosynthetic Gene Cluster). V-shapes represent the BGCs clustered with validated BGCs in the MIBiG database. "Novel" indicates compounds in the antiSMASH annotation results that have a similarity of less than 30% with most known compounds. (b).Examples are provided to show five BGC clusters that exhibit high similarity with the reference BGC in the *Streptomyces globisporus_A* strain.

Figure S7. Cellulose-degrading-related CAZymes distribution in the YFR. The heatmap illustrates the presence and absence of cellulose-degrading related CAZymes within the strains.

Figure S8. Sole Carbon Source Utilization Assays for Bacterial Species. Heatmap depicting the normalized growth of 50 aerobic bacterial strains across three phyla on six polysaccharide substrates (Arabinogalactan, Cellulose, Fructan, Pectin, Starch, Xylan). The color gradient represents Normalized Growth, scaled from 0 (light yellow) to 1.00 (dark green), derived from baseline-corrected OD600 values (subtracting Time=0 OD, with values <0.06 set to 0). Strains with absorbance increase >0.1 are marked with an asterisk (*).

Figure S9. Quality distribution of virus genomes. The completeness and contamination status of the 459 virus genomes. Quality evaluations and specific values are derived from CheckV annotations, with green representing complete viral genomes, blue indicating high-quality viral genomes, and red indicating genomes of medium quality.

Figure S10. The number of bacteriophages carried by strains from different phyla.

Figure S11. Distribution of KOs corresponding to the viral genome population in the host.

Figure S12. Folate Biosynthesis and ABC Transporters Pathways with Viral Genome. The viral genomes provided by strain T2106134295 and strain T2108178176 were mapped to the Folate biosynthesis and ABC transporters pathways using KO mapping. The red arrows indicate that these key enzymes are encoded by the viral genomes.

Figure S1

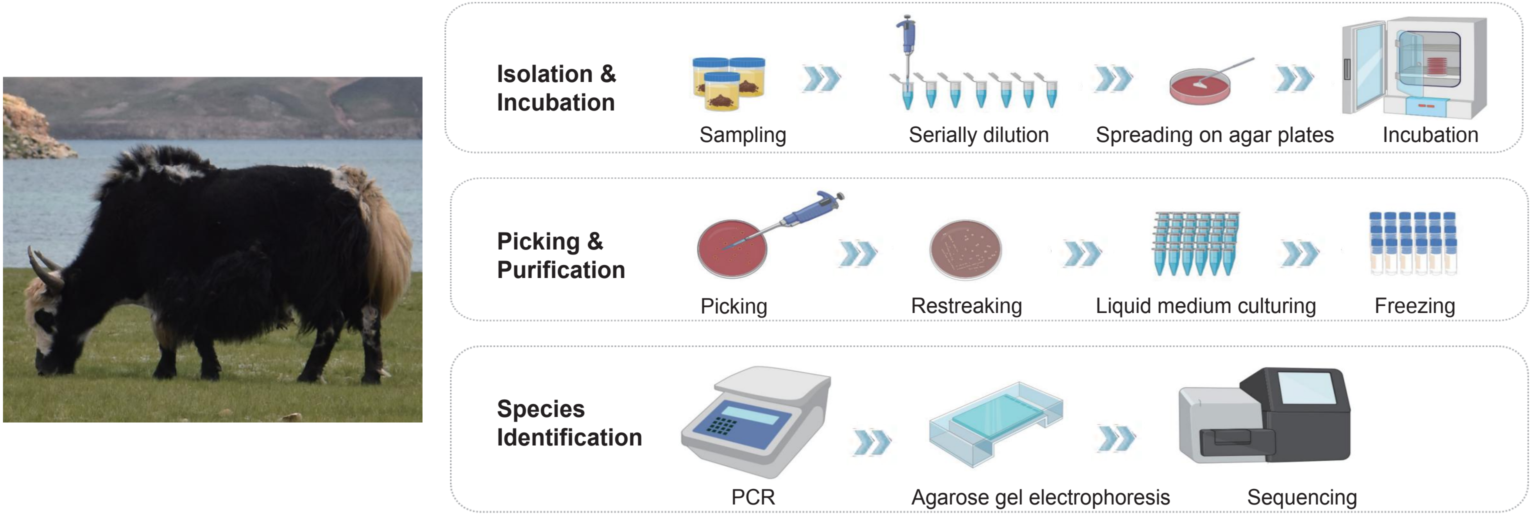
Isolation & Incubation
Sampling
Serially dilution
Spreading on agar plates
Incubation
Picking & Purification
Picking
Restreaking
Liquid medium culturing
Freezing
Species Identification
PCR
Agarose gel electrophoresis
Sequencing

Figure S2

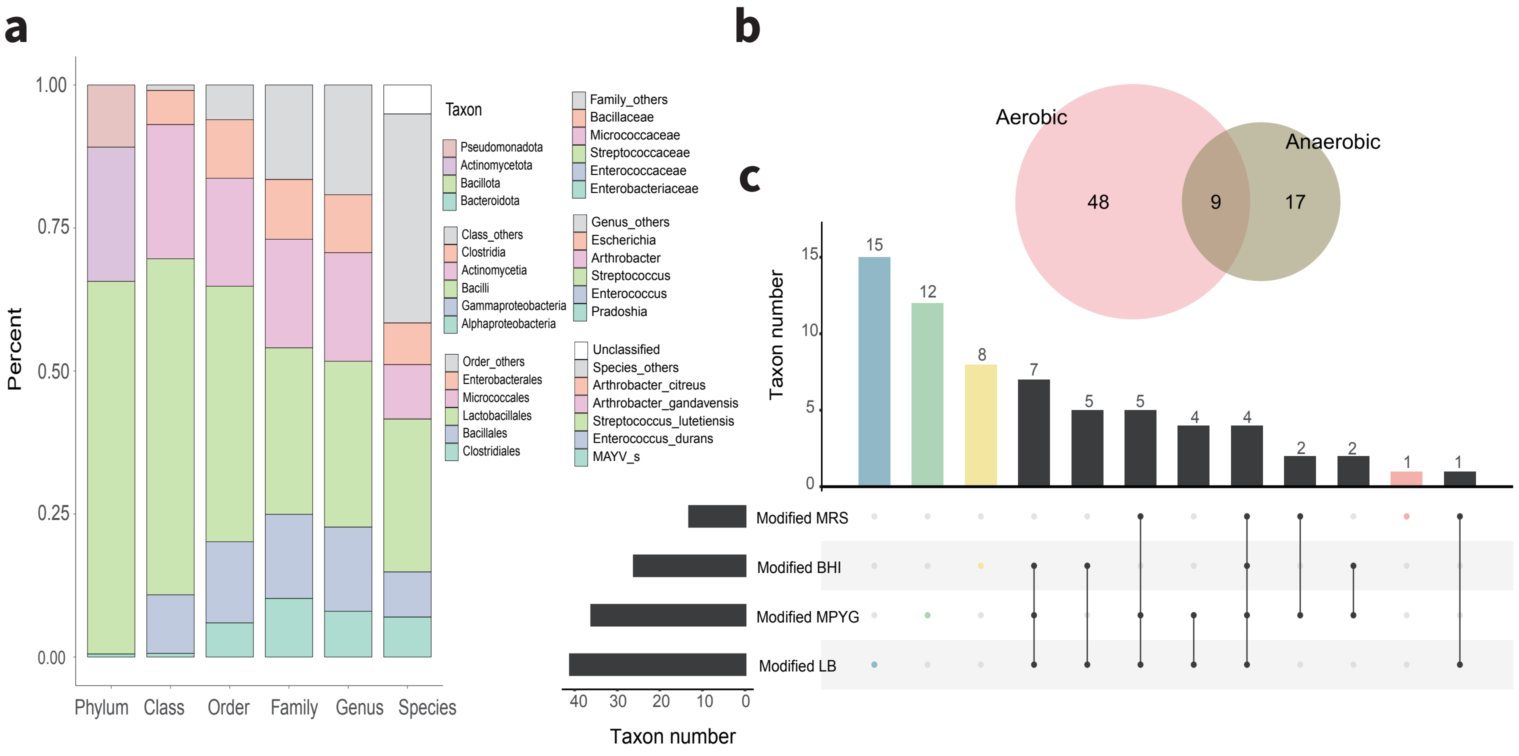
a
b
c
1.00
0.75
0.50
0.25
0.00
Percent
Phylum
Class
Order
Family
Genus
Species
Taxon
Pseudomonadota
Actinomycetota
Bacillota
Bacteroidota
Class_others
Clostridia
Actinomycetia
Bacilli
Gammaproteobacteria
Alphaproteobacteria
Order_others
Enterobacterales
Micrococcales
Lactobacillales
Bacillales
Clostridiales
Family_others
Bacillaceae
Micrococcaceae
Streptococcaceae
Enterococcaceae
Enterobacteriaceae
Genus_others
Escherichia
Arthrobacter
Streptococcus
Enterococcus
Pradoshia
Unclassified
Species_others
Arthrobacter_citreus
Arthrobacter_gandavensis
Streptococcus_lutetiensis
Enterococcus_durans
MAYV_s
Aerobic
Anaerobic
48
9
17
Taxon number
15
12
8
7
5
5
4
4
2
2
1
1
Modified MRS
Modified BHI
Modified MPYG
Modified LB
40 30 20 10 0
Taxon number

Figure S3

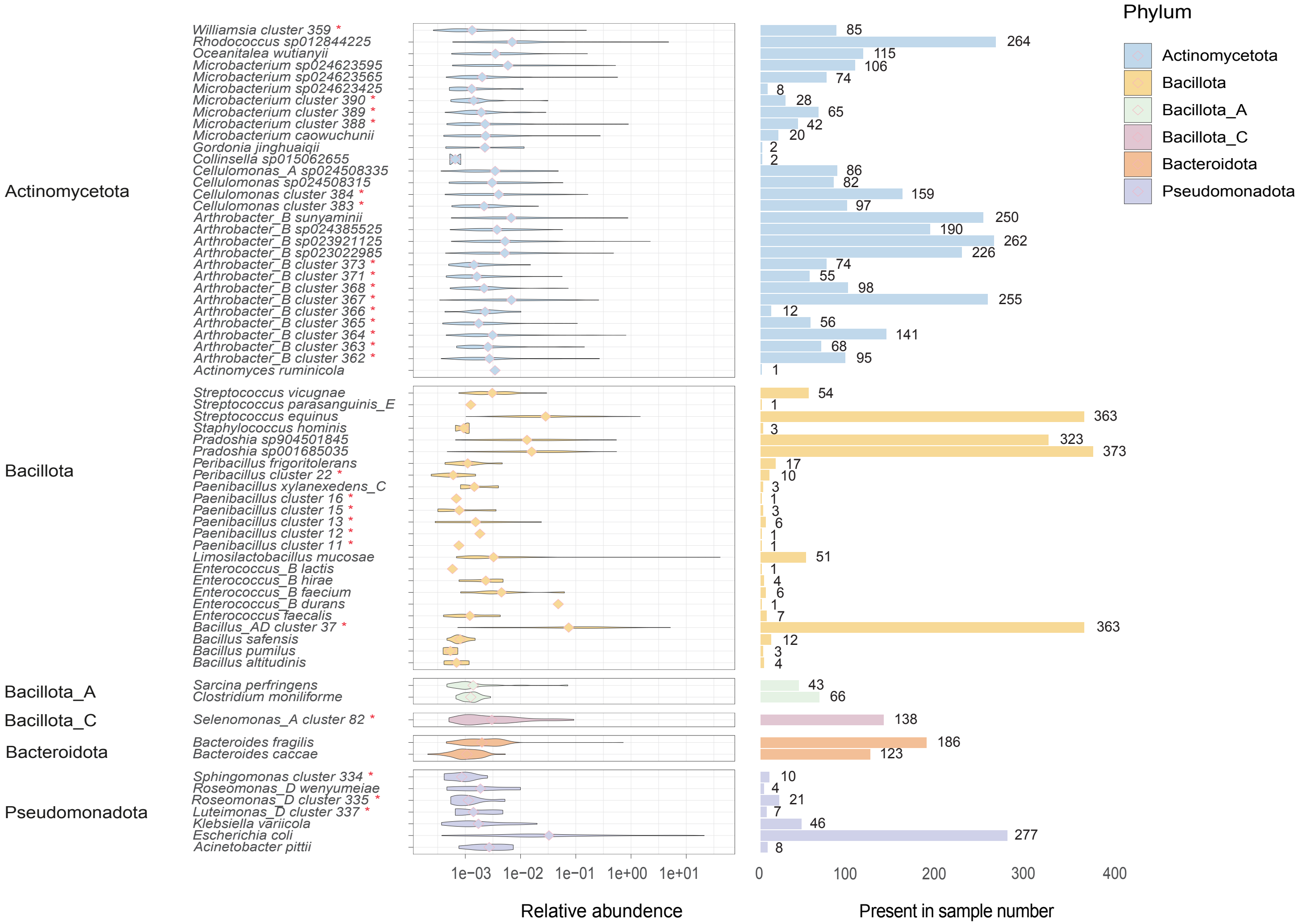

Phylum
Actinomycetota
Bacillota
Bacillota_A
Bacillota_C
Bacteroidota
Pseudomonadota
Williamsia cluster 359 *
Rhodococcus sp012844225
Oceanitalea wutianyii
Microbacterium sp024623595
Microbacterium sp024623565
Microbacterium sp024623425
Microbacterium cluster 390 *
Microbacterium cluster 389 *
Microbacterium cluster 388 *
Microbacterium caowuchunii
Gordonia jinghuaiqii
Collinsella sp015062655
Cellulomonas_A sp024508335
Cellulomonas sp024508315
Cellulomonas cluster 384 *
Cellulomonas cluster 383 *
Arthrobacter_B sunyaminii
Arthrobacter_B sp024385525
Arthrobacter_B sp023921125
Arthrobacter_B sp023022985
Arthrobacter_B cluster 373 *
Arthrobacter_B cluster 371 *
Arthrobacter_B cluster 368 *
Arthrobacter_B cluster 367 *
Arthrobacter_B cluster 366 *
Arthrobacter_B cluster 365 *
Arthrobacter_B cluster 364 *
Arthrobacter_B cluster 363 *
Arthrobacter_B cluster 362 *
Actinomyces ruminicola
Streptococcus vicugnae
Streptococcus parasanguinis_E
Streptococcus equinus
Staphylococcus hominis
Pradoshia sp904501845
Pradoshia sp001685035
Peribacillus frigoritolerans
Peribacillus cluster 22 *
Paenibacillus xylanexedens_C
Paenibacillus cluster 16 *
Paenibacillus cluster 15 *
Paenibacillus cluster 13 *
Paenibacillus cluster 12 *
Paenibacillus cluster 11 *
Limosilactobacillus mucosae
Enterococcus_B lactis
Enterococcus_B hirae
Enterococcus_B faecium
Enterococcus_B durans
Enterococcus faecalis
Bacillus_AD cluster 37 *
Bacillus safensis
Bacillus pumilus
Bacillus altitudinis
Sarcina perfringens
Clostridium moniliforme
Selenomonas_A cluster 82 *
Bacteroides fragilis
Bacteroides caccae
Sphingomonas cluster 334 *
Roseomonas_D wenyumeiae
Roseomonas_D cluster 335 *
Luteimonas_D cluster 337 *
Klebsiella variicola
Escherichia coli
Acinetobacter pittii
85
264
115
106
74
8
28
65
42
20
2
2
86
82
159
97
250
190
262
226
74
55
98
255
12
56
141
68
95
1
54
1
363
3
323
373
17
10
3
1
3
6
1
1
51
1
4
6
1
7
363
12
3
4
43
66
138
186
123
10
4
21
7
46
277
8
1e−03
1e−02
1e−01
1e+00
1e+01
Relative abundence
0
100
200
300
400
Present in sample number

Figure S4

a

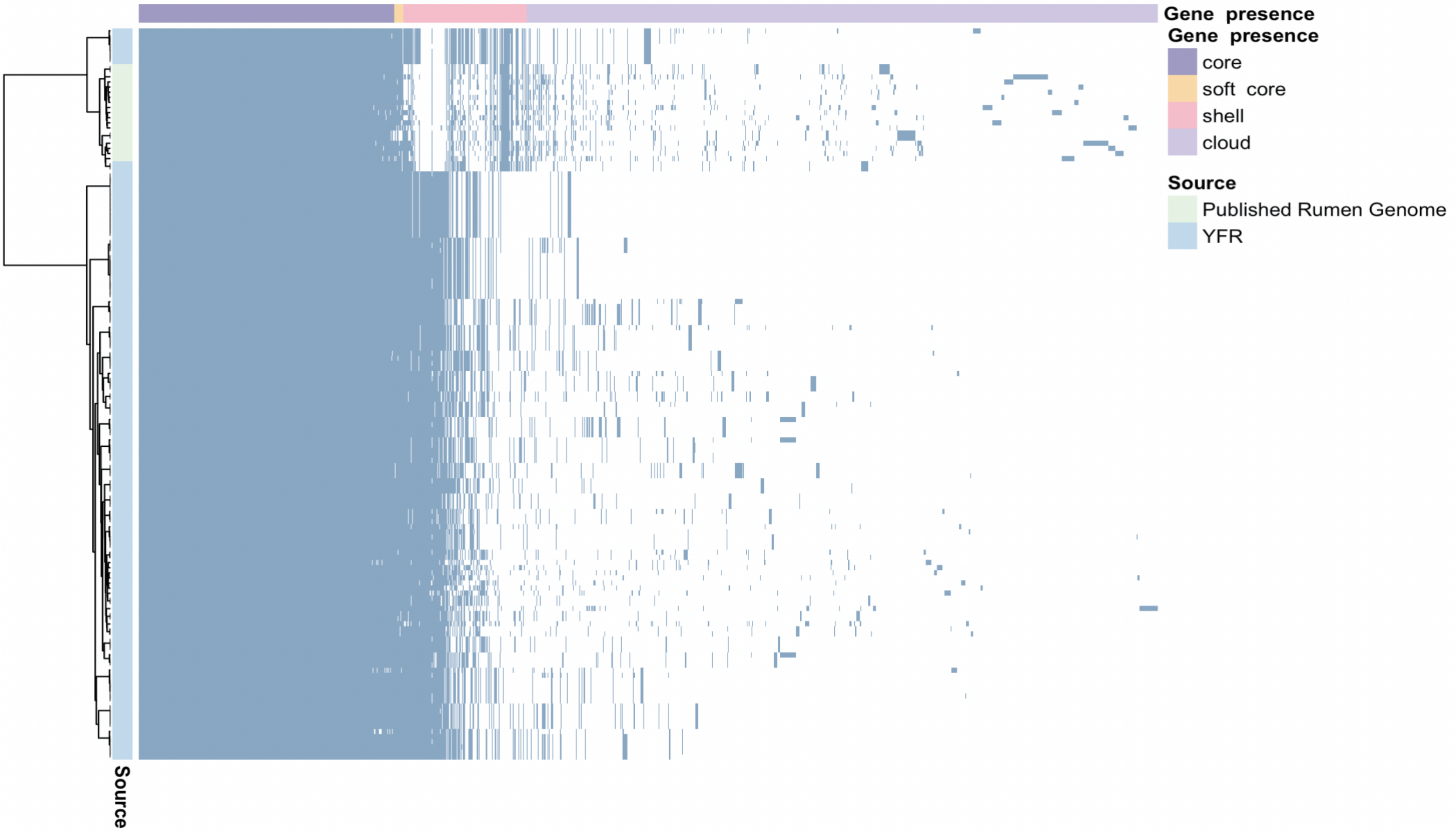
Gene presence
Gene presence
core
soft core
shell
cloud
Source
Published Rumen Genome
YFR
Source

b

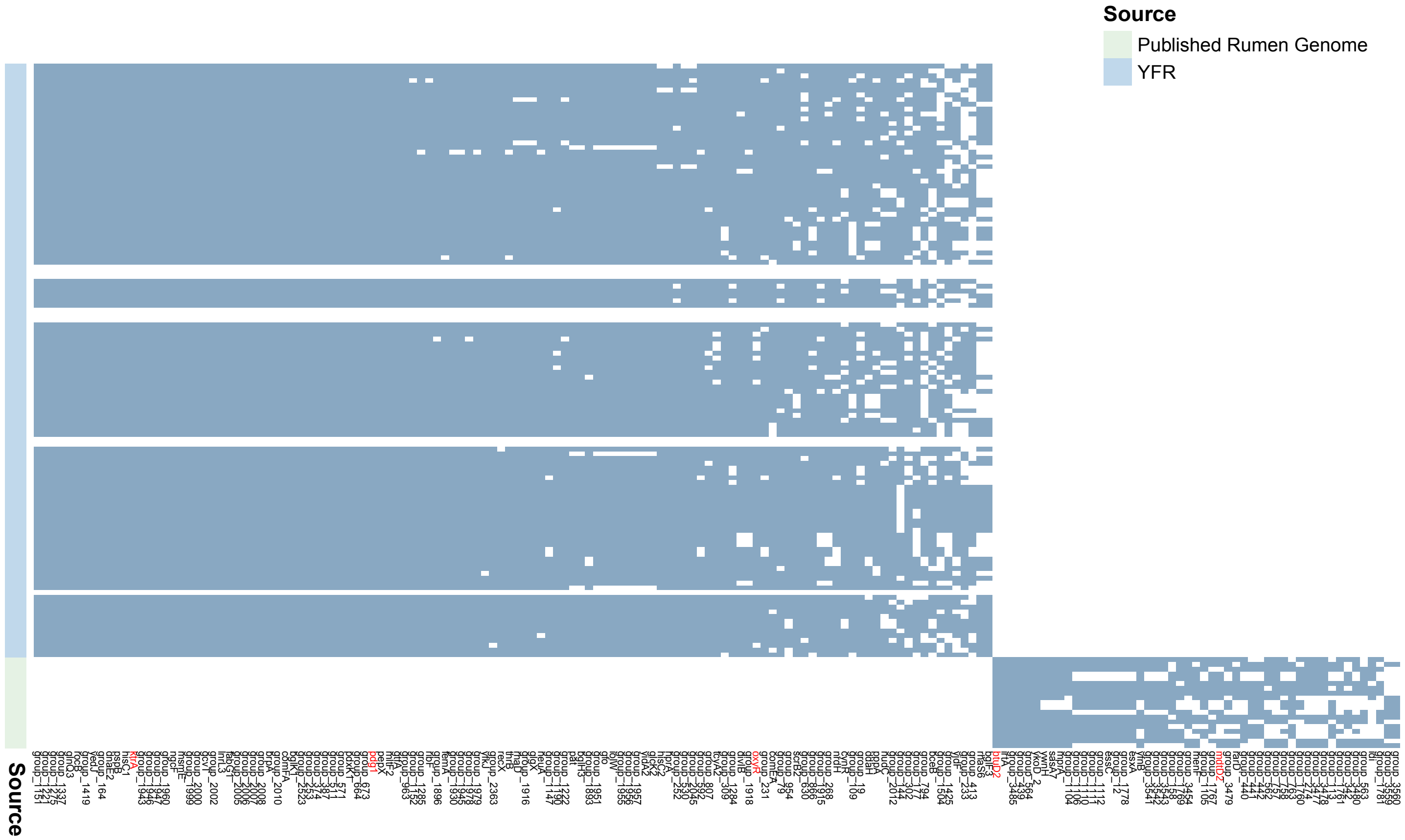
Source
Published Rumen Genome
YFR
Source

Figure S5

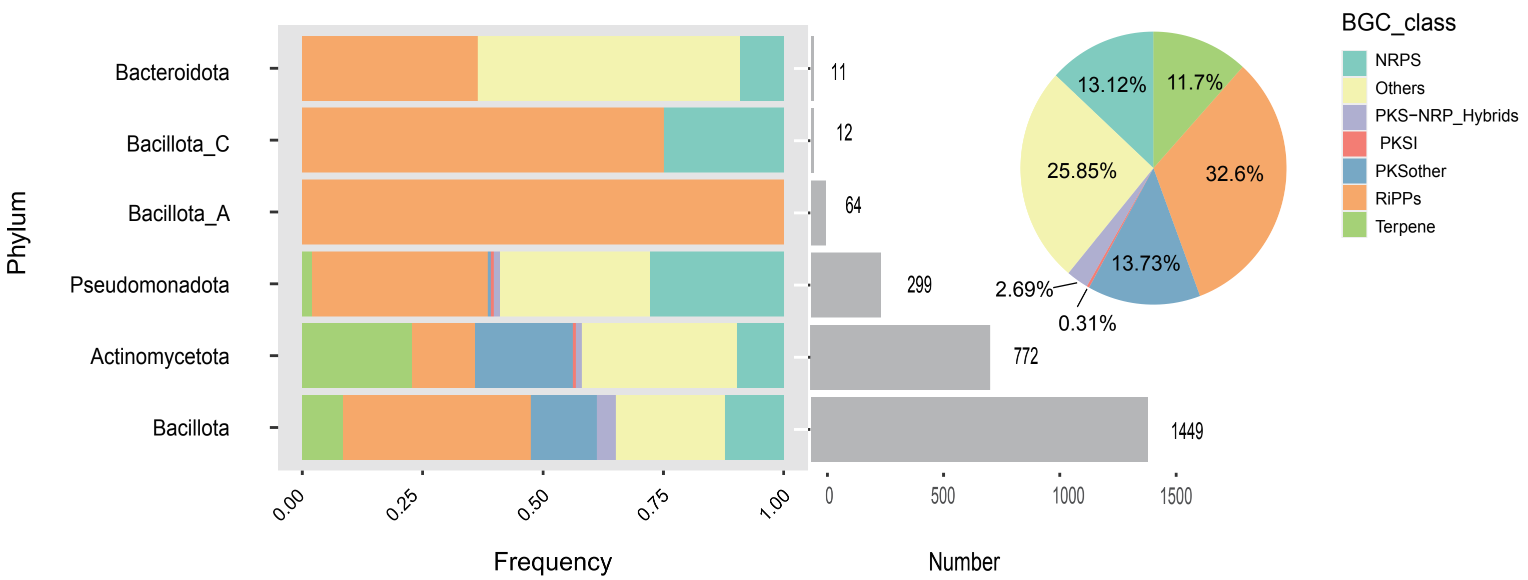

Phylum
Bacteroidota
Bacillota_C
Bacillota_A
Pseudomonadota
Actinomycetota
Bacillota
0.00
0.25
0.50
0.75
1.00
Frequency
11
12
64
299
772
1449
0
500
1000
1500
Number
13.12%
11.7%
25.85%
32.6%
13.73%
2.69%
0.31%
BGC_class
NRPS
Others
PKS−NRP_Hybrids
PKSI
PKSother
RiPPs
Terpene

Figure S6

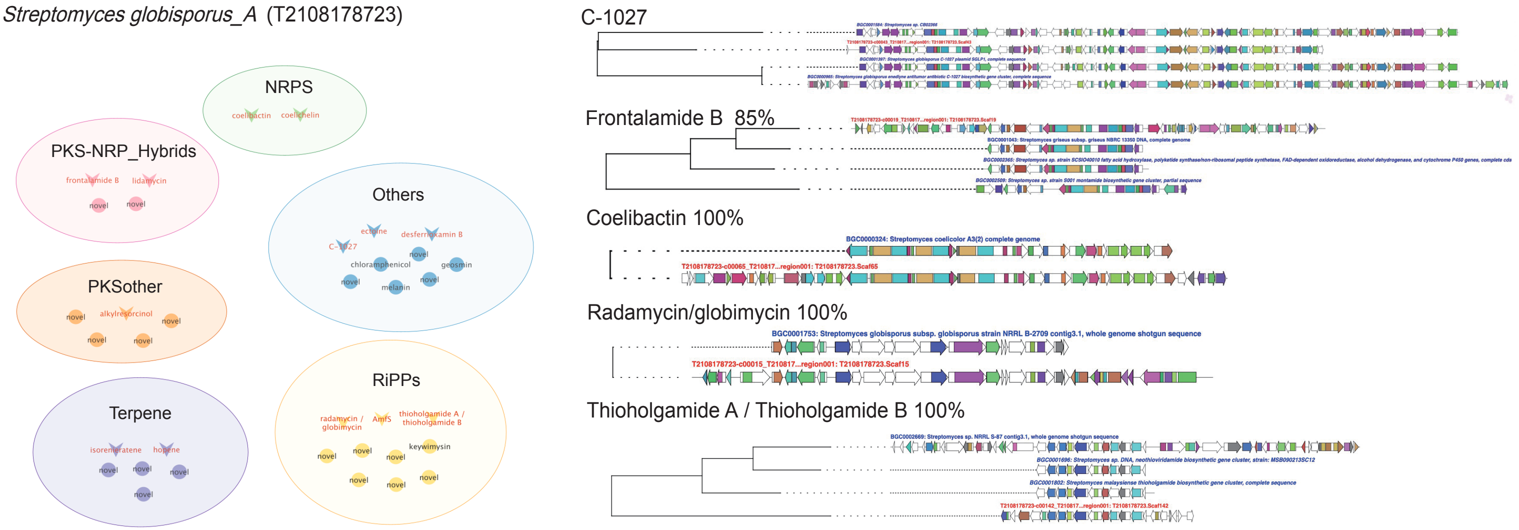

Streptomyces globisporus_A (T2108178723)
NRPS
coelibactin
coelichelin
PKS-NRP_Hybrids
frontalamide B
lidamycin
novel
novel
Others
ectoine
desferrioxamin B
C-1027
novel
chloramphenicol
geosmin
novel
melanin
novel
PKSother
novel
alkylresorcinol
novel
novel
novel
RiPPs
radamycin / globimycin
AmfS
thioholgamide A / thioholgamide B
keywimysin
novel
novel
novel
novel
novel
novel
Terpene
isorenieratene
hopene
novel
novel
novel
novel
C-1027
Frontalamide B 85%
Coelibactin 100%
BGC0000324: Streptomyces coelicolor A3(2) complete genome
T2108178723-c00065_T210817...region001: T2108178723.Scaf65
Radamycin/globimycin 100%
BGC0001753: Streptomyces globisporus subsp. globisporus strain NRRL B-2709 contig3.1, whole genome shotgun sequence
T2108178723-c00015_T210817...region001: T2108178723.Scaf15
Thioholgamide A / Thioholgamide B 100%

Figure S7

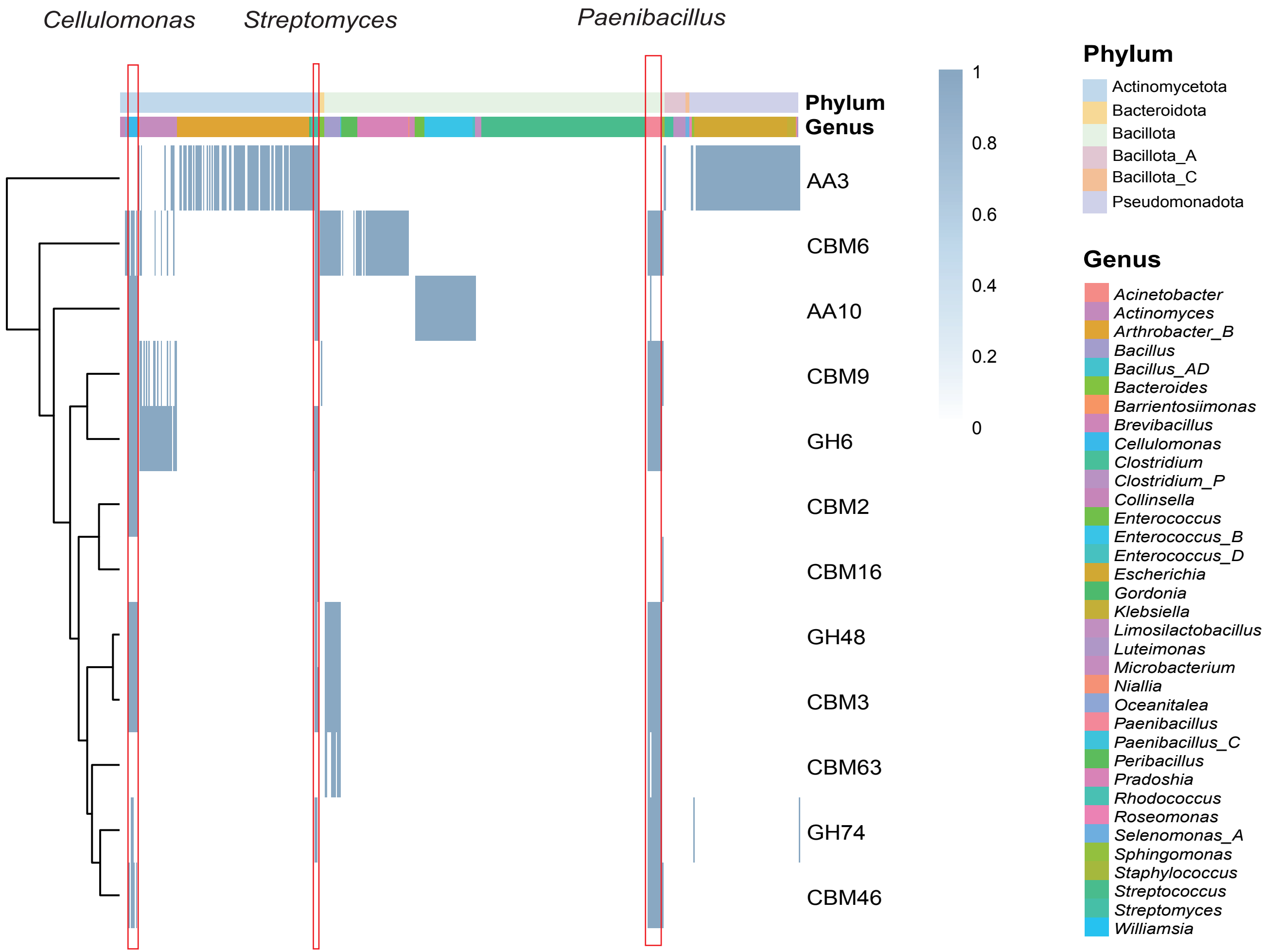
Cellulomonas
Streptomyces
Paenibacillus
Phylum
Genus
AA3
CBM6
AA10
CBM9
GH6
CBM2
CBM16
GH48
CBM3
CBM63
GH74
CBM46
1
0.8
0.6
0.4
0.2
0
Phylum
Actinomycetota
Bacteroidota
Bacillota
Bacillota_A
Bacillota_C
Pseudomonadota
Genus
Acinetobacter
Actinomyces
Arthrobacter_B
Bacillus
Bacillus_AD
Bacteroides
Barrientosiimonas
Brevibacillus
Cellulomonas
Clostridium
Clostridium_P
Collinsella
Enterococcus
Enterococcus_B
Enterococcus_D
Escherichia
Gordonia
Klebsiella
Limosilactobacillus
Luteimonas
Microbacterium
Niallia
Oceanitalea
Paenibacillus
Paenibacillus_C
Peribacillus
Pradoshia
Rhodococcus
Roseomonas
Selenomonas_A
Sphingomonas
Staphylococcus
Streptococcus
Streptomyces
Williamsia

Figure S8

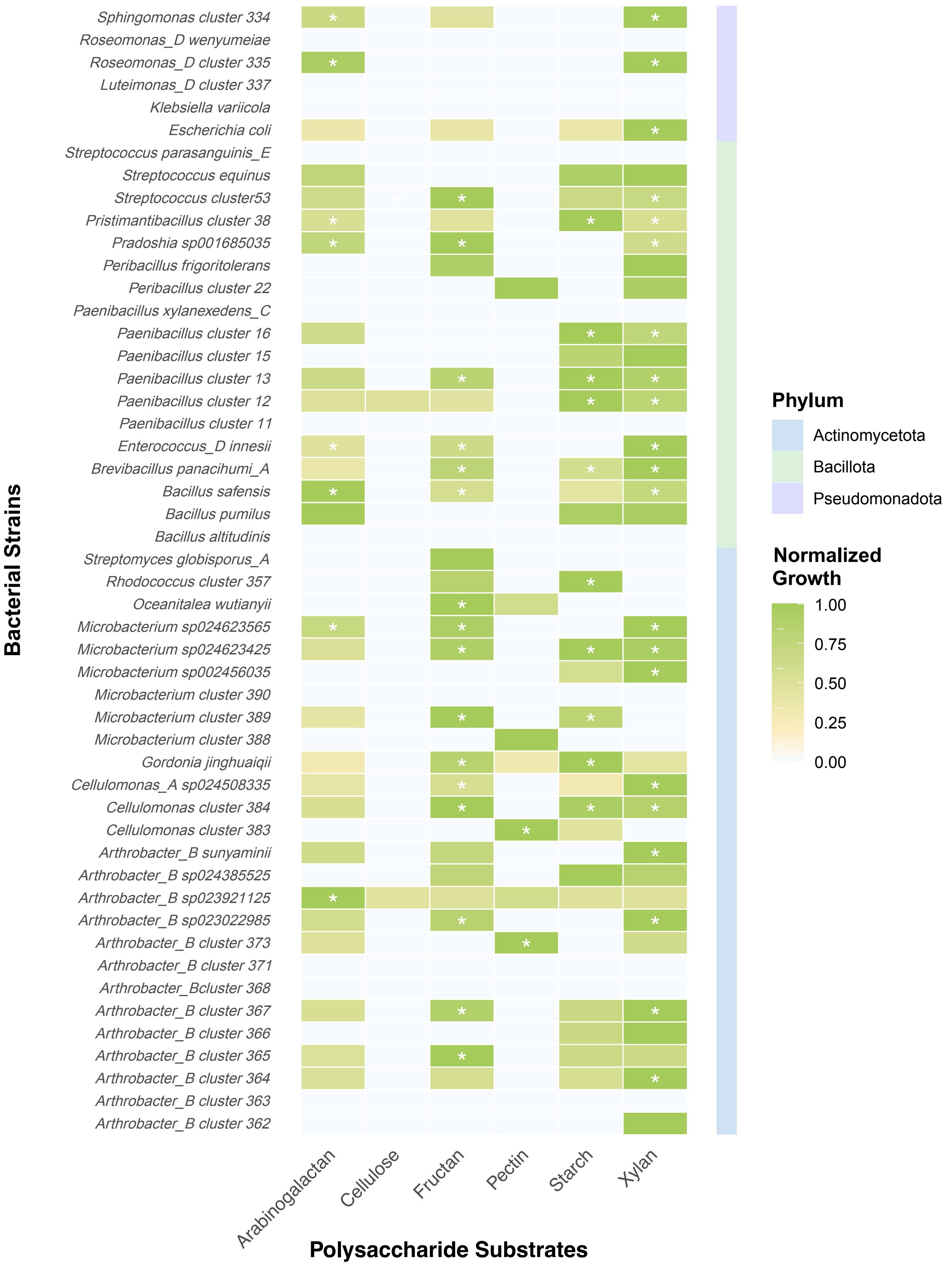

Sphingomonas cluster 334
Roseomonas_D wenyumeiae
Roseomonas_D cluster 335
Luteimonas_D cluster 337
Klebsiella variicola
Escherichia coli
Streptococcus parasanguinis_E
Streptococcus equinus
Streptococcus cluster53
Pristimantibacillus cluster 38
Pradoshia sp001685035
Peribacillus frigoritolerans
Peribacillus cluster 22
Paenibacillus xylanexedens_C
Paenibacillus cluster 16
Paenibacillus cluster 15
Paenibacillus cluster 13
Paenibacillus cluster 12
Paenibacillus cluster 11
Enterococcus_D innesii
Brevibacillus panacihumi_A
Bacillus safensis
Bacillus pumilus
Bacillus altitudinis
Streptomyces globisporus_A
Rhodococcus cluster 357
Oceanitalea wutianyii
Microbacterium sp024623565
Microbacterium sp024623425
Microbacterium sp002456035
Microbacterium cluster 390
Microbacterium cluster 389
Microbacterium cluster 388
Gordonia jinghuaiqii
Cellulomonas_A sp024508335
Cellulomonas cluster 384
Cellulomonas cluster 383
Arthrobacter_B sunyaminii
Arthrobacter_B sp024385525
Arthrobacter_B sp023921125
Arthrobacter_B sp023022985
Arthrobacter_B cluster 373
Arthrobacter_B cluster 371
Arthrobacter_Bcluster 368
Arthrobacter_B cluster 367
Arthrobacter_B cluster 366
Arthrobacter_B cluster 365
Arthrobacter_B cluster 364
Arthrobacter_B cluster 363
Arthrobacter_B cluster 362
Bacterial Strains
Arabinogalactan
Cellulose
Fructan
Pectin
Starch
Xylan
Polysaccharide Substrates
Phylum
Actinomycetota
Bacillota
Pseudomonadota
Normalized Growth
1.00
0.75
0.50
0.25
0.00

Figure S9

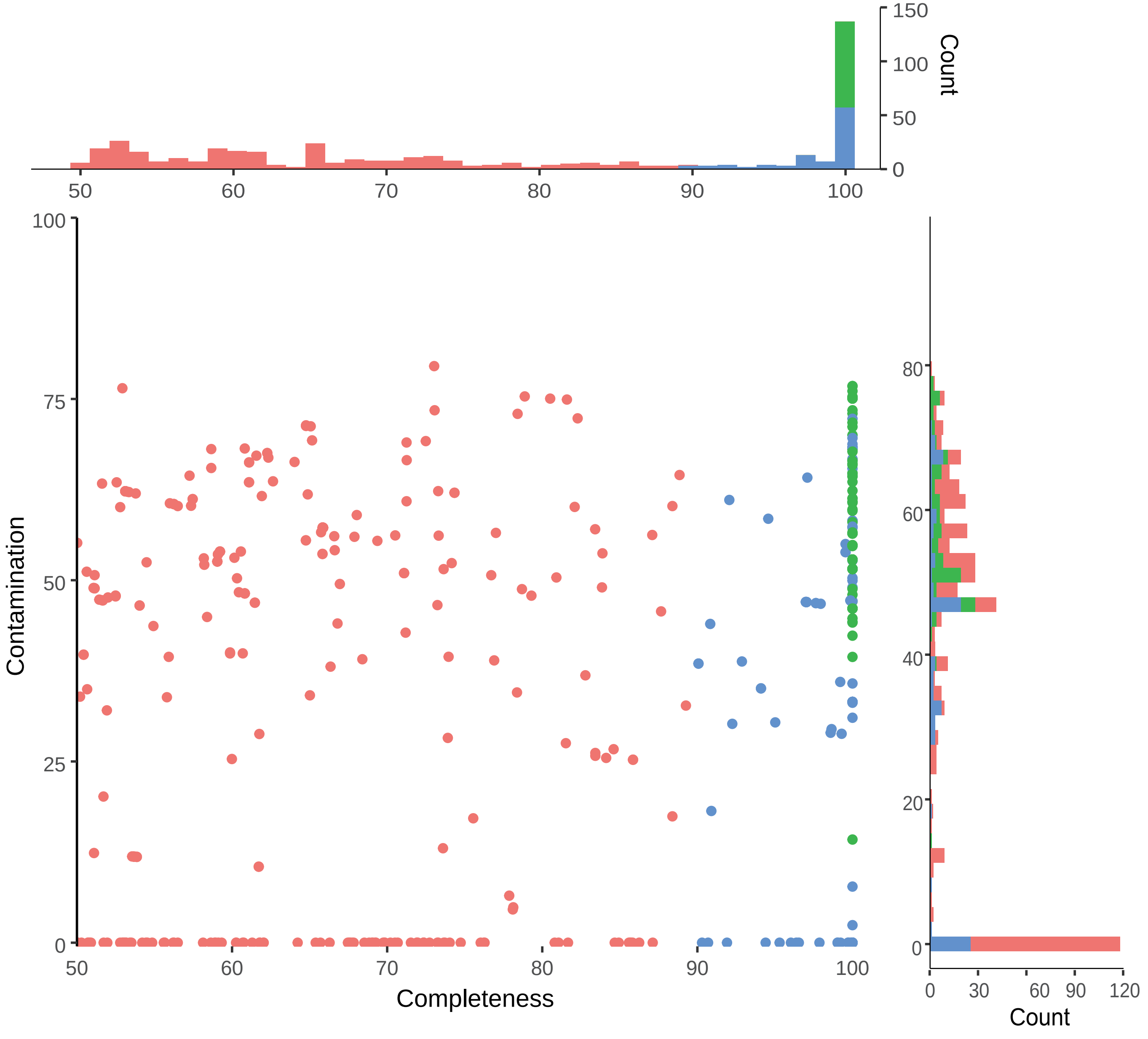
Count
150
100
50
0
50
60
70
80
90
100
Contamination
Completeness
Count
0
30
60
90
120
25
75
20
40
80

Figure S10

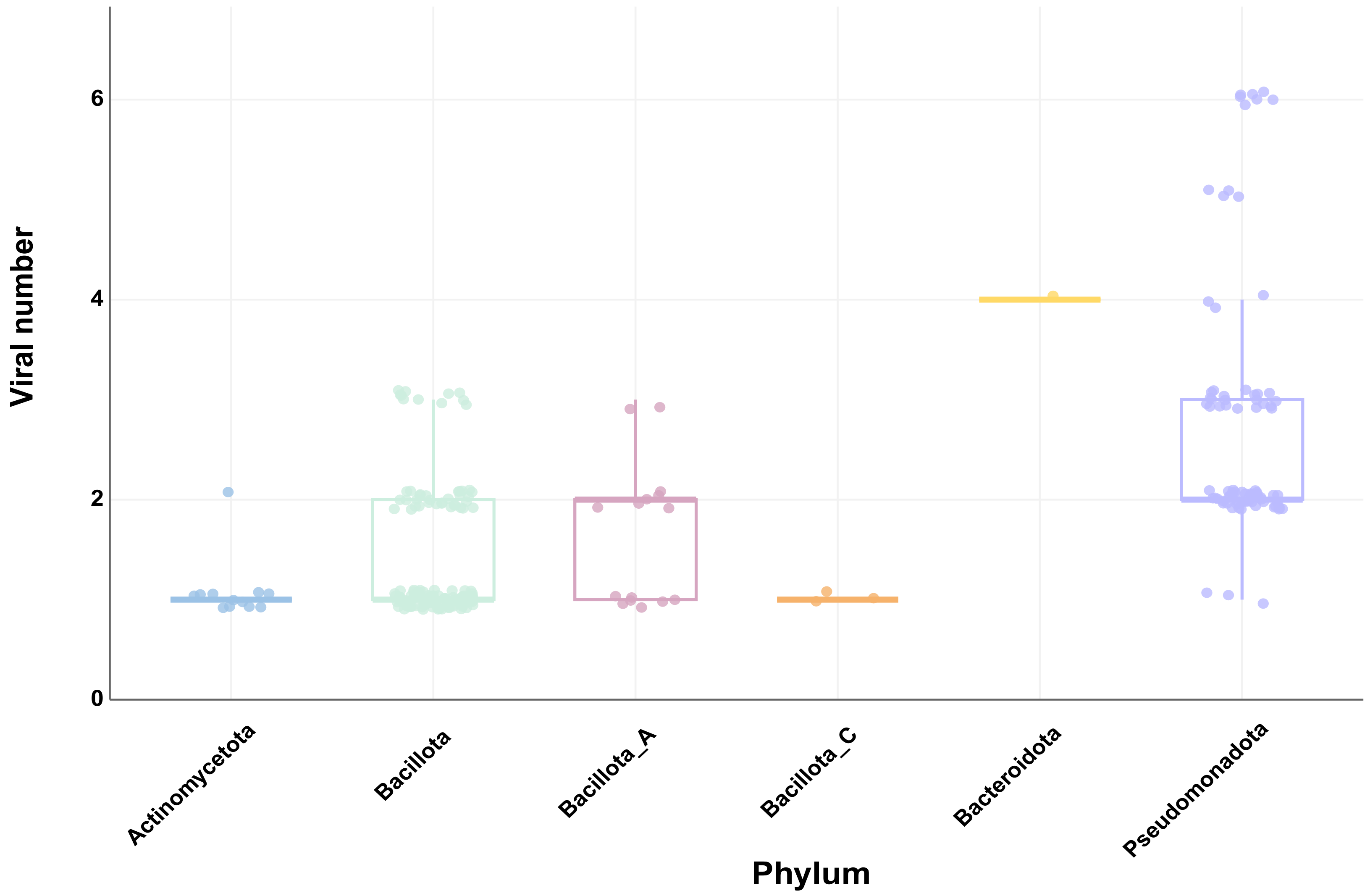

Viral number
6
4
2
0
Actinomycetota
Bacillota
Bacillota_A
Bacillota_C
Bacteroidota
Pseudomonadota
Phylum

Figure S11

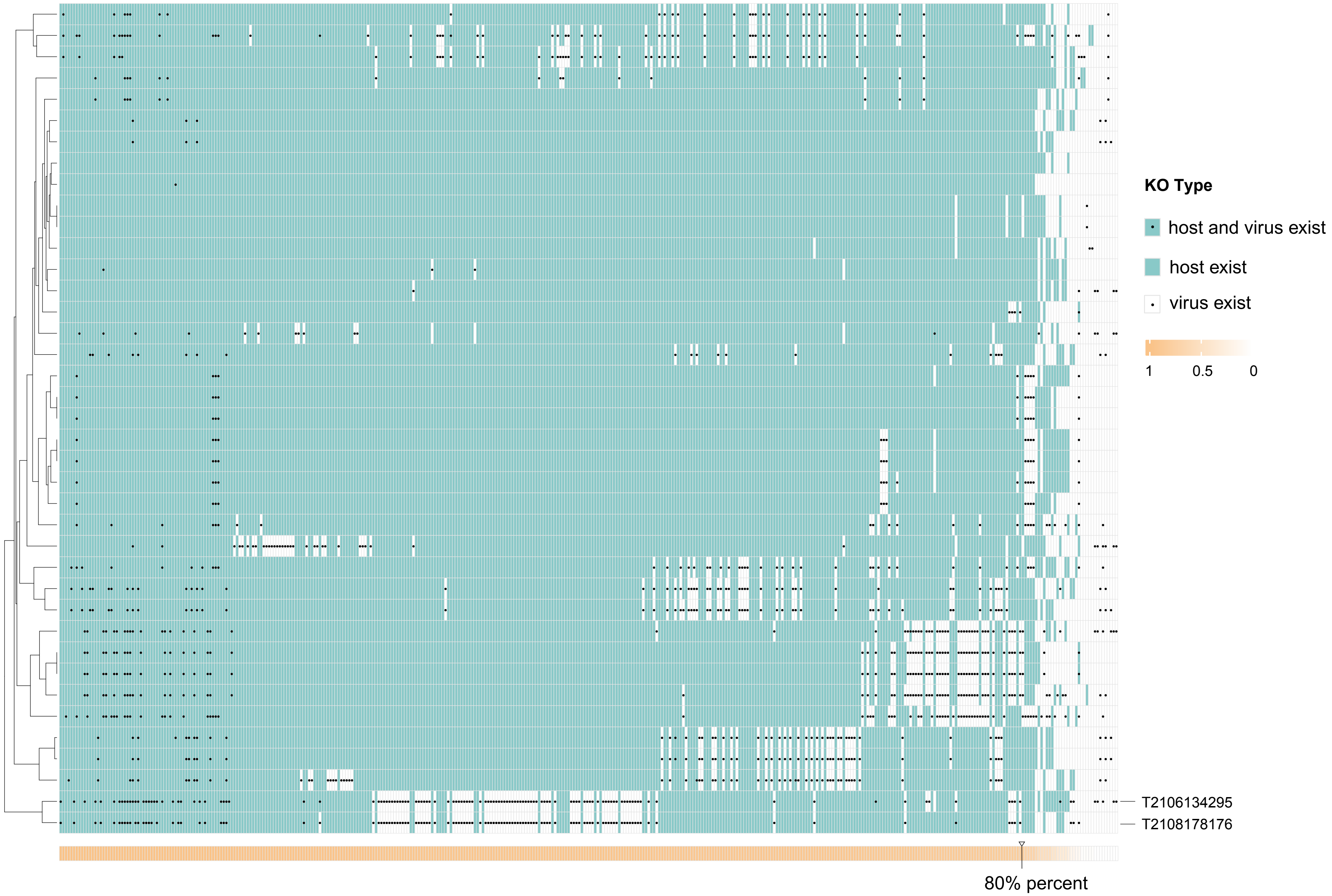

KO Type
host and virus exist
host exist
virus exist
1
0.5
0
T2106134295
T2108178176
80% percent

Figure S12

Folate biosynthesis

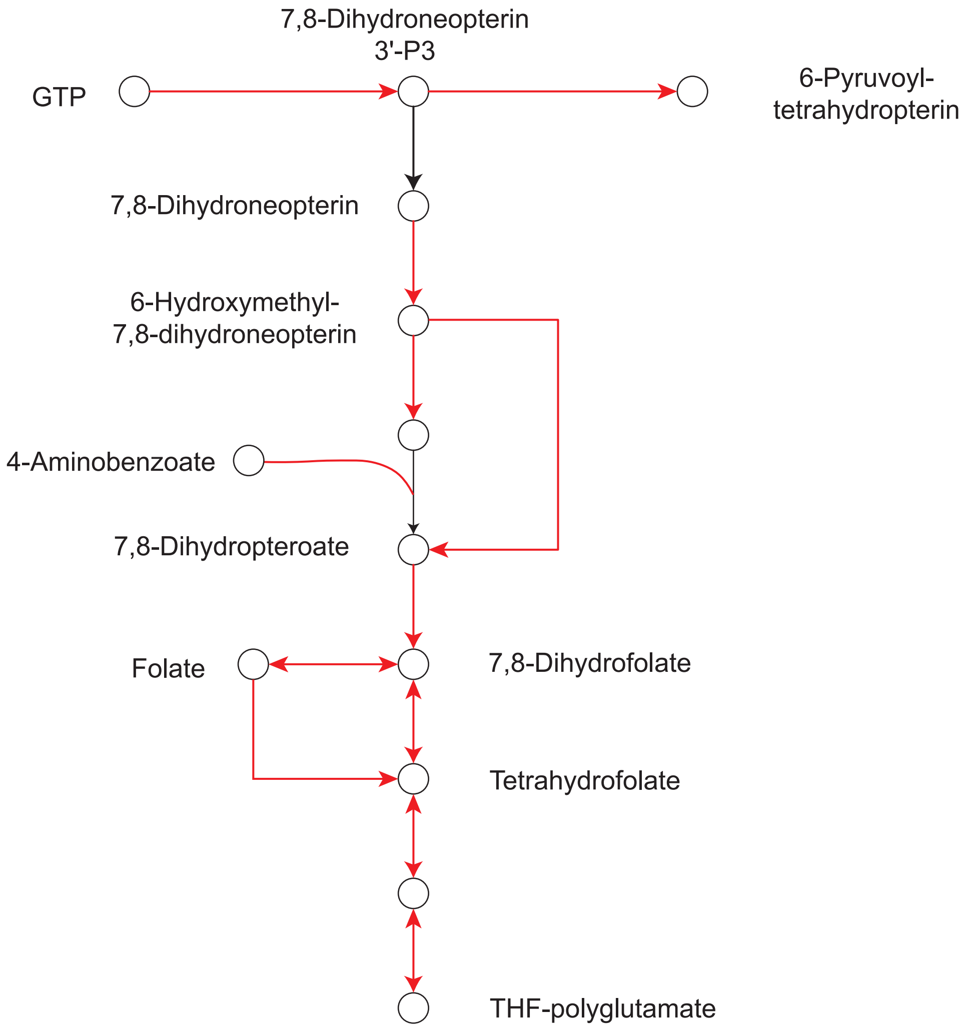

ABC transporters

Mineral and organic ion transporters :

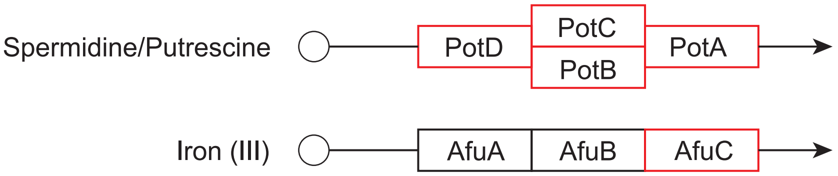

Phosphate and amino acid transporters :

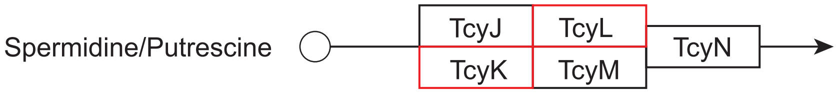

Supplement: Supplemental material — Supplemental information and figures. [file msystems.00367-25-s0001.pdf]
